# Establishment and analysis of a reference transcriptome for *Spodoptera frugiperda*

**DOI:** 10.1186/1471-2164-15-704

**Published:** 2014-08-23

**Authors:** Fabrice Legeai, Sylvie Gimenez, Bernard Duvic, Jean-Michel Escoubas, Anne-Sophie Gosselin Grenet, Florence Blanc, François Cousserans, Imène Séninet, Anthony Bretaudeau, Doriane Mutuel, Pierre-Alain Girard, Christelle Monsempes, Ghislaine Magdelenat, Frédérique Hilliou, René Feyereisen, Mylène Ogliastro, Anne-Nathalie Volkoff, Emmanuelle Jacquin-Joly, Emmanuelle d’Alençon, Nicolas Nègre, Philippe Fournier

**Affiliations:** INRA, UMR Institut de Génétique, Environnement et Protection des Plantes (IGEPP), BioInformatics Platform for Agroecosystems Arthropods (BIPAA), Campus Beaulieu, Rennes, France; INRIA, IRISA, Genscale, Campus Beaulieu, Rennes, France; INRA, UMR1333, DGIMI, Montpellier, France; UMR1333, DGIMI, Université Montpellier 2, Montpellier, France; INRIA, IRISA, Genouest, Campus Beaulieu, Rennes, France; INRA, UMR 1392, Institut d’Ecologie et des Sciences de l’Environnement de Paris (iEES-Paris), Versailles, France; Genoscope, Evry, France; INRA - CNRS - Univ.Nice Sophia Antipolis, UMR Institut Sophia Agrobiotech (ISA), Sophia Antipolis, France; Institut Universitaire de France (IUF), Paris, France

**Keywords:** *Spodoptera frugiperda*, Transcriptomics, Immunity, Olfaction

## Abstract

**Background:**

*Spodoptera frugiperda* (Noctuidae) is a major agricultural pest throughout the American continent. The highly polyphagous larvae are frequently devastating crops of importance such as corn, sorghum, cotton and grass. In addition, the Sf9 cell line, widely used in biochemistry for *in vitro* protein production, is derived from *S. frugiperda* tissues. Many research groups are using *S. frugiperda* as a model organism to investigate questions such as plant adaptation, pest behavior or resistance to pesticides.

**Results:**

In this study, we constructed a reference transcriptome assembly (Sf_TR2012b) of RNA sequences obtained from more than 35 *S. frugiperda* developmental time-points and tissue samples. We assessed the quality of this reference transcriptome by annotating a ubiquitous gene family - ribosomal proteins - as well as gene families that have a more constrained spatio-temporal expression and are involved in development, immunity and olfaction. We also provide a time-course of expression that we used to characterize the transcriptional regulation of the gene families studied.

**Conclusion:**

We conclude that the Sf_TR2012b transcriptome is a valid reference transcriptome. While its reliability decreases for the detection and annotation of genes under strong transcriptional constraint we still recover a fair percentage of tissue-specific transcripts. That allowed us to explore the spatial and temporal expression of genes and to observe that some olfactory receptors are expressed in antennae and palps but also in other non related tissues such as fat bodies. Similarly, we observed an interesting interplay of gene families involved in immunity between fat bodies and antennae.

**Electronic supplementary material:**

The online version of this article (doi:10.1186/1471-2164-15-704) contains supplementary material, which is available to authorized users.

## Background

Many organisms of major importance in economy and health of human populations are non-model organisms and thus lack efficient genetic resources that could be used to speed up and facilitate the work of research groups throughout the world. However, the advent of Next Generation Sequencing (NGS), by decreasing sequencing costs of a factor 1,000 [1], provided the opportunity to sequence the genomes of new organisms (new-models) at different stages of completion. In general, obtaining complete genome sequences for a given organism is immediately followed by the computational and manual annotation of its gene catalog. Genes, in the sense of protein-coding genes, are the major focus of most genome sequencing consortia. Thus, obtaining first a complete transcriptome for an organism, might, in most cases, cover the needs of a specific scientific community. Furthermore, obtaining a good quality reference transcriptome as a first step of a genome sequence project could prove immensely beneficial for gene prediction and annotation.

The Lepidoptera *Spodoptera frugiperda* (Noctuidae) is an intensely studied organism, yet lacking a comprehensive genomic resource. *S. frugiperda*, also known as the Fall Army Worm (FAW) is a noctuid moth, classified as a major crop pest by USDA, INRA and other national agronomic agencies. In the United States and in Brazil, it is a threat to corn but is also found devastating cotton, sorghum and other grass-like crops such as rice [2, 3]. Its area of distribution concerns almost the entirety of the American continent [3], thus much work has been devoted to study the biology of this insect. In order to improve the tools at our disposal to efficiently understand the biology of *S. frugiperda*, we report here the generation of an NGS-based sequencing of RNA libraries obtained from a large variety and number of tissues and developmental time-points. These libraries have been assembled together in a reference transcriptome, dubbed Sf_TR2012b, comprising around 55,000 sequences available for search through a dedicated database. We further used the Sf_TR2012b assembly to annotate several gene families and study their expression profile. We focused especially on the genes involved in immunity and in olfaction. We validated some of our developmental genes predictions by qPCR to demonstrate that in Sf_TR2012b, some regulated transcripts without clear orthology are *bona fide* functional genes in *S. frugiperda*.

## Results and discussion

### Construction of a reference transcriptome

The reference transcriptome presented here is named Sf_TR2012b. We incorporated various sources of RNA sequences from 454, Illumina and Sanger sequencing and had to develop a custom pipeline. This assembly is described in details in the Methods section and illustrated in Additional file 1: Figure S1. Its assembly has been performed by the software MIRA [4] and incorporated various sources of RNA sequences obtained from a laboratory strain of *S. frugiperda*. The main source of RNA molecules has been obtained by 454 RNAseq of a library containing cDNA extracted from 27 different samples (Table 1) comprising 14 developmental time-points samples (Table 1, column A) and 13 dissected tissues (Table 1, column B), in order to cover the majority of the mRNA produced by the FAW. First these 454 sequences have been assembled in ~183,000 different clusters, referred to in here, as Sf_GATC_Clusters (Table 2). In a subsequent assembly step (see Methods), Sf_GATC_Clusters have been used as input, as well as 10 RNA samples (Table 1, column C), sequenced using the Illumina technology [5]. A collection of Expressed Sequence Tags (ESTs), previously described and available through Spodobase (http://bioweb.ensam.inra.fr/spodobase/) [6], was also included in the final assembly. The final Sf_TR2012b assembly comprises ~55,000 sequences for a total of ~37 M nt (Table 2 and Additional file 2: Table S1).

**Table 1:** **Biological samples from which RNA has been extracted for the construction of the reference transcriptome**

454 RNA sequencing		Illumina RNA sequencing	
A. 14 Developmental time-points	B. 13 Dissected tissues	C. 10 samples sequenced by Illumina	D. Abbreviations of the libraries
Eggs	Male adults antennae	Developping eggs	Eggs
Developing eggs*	Female adults antennae	L2 larvae (early stage)	L2e
L1 larvae	Larvae antennae	L2 larvae (late stage)	L2l
L2 larvae (early stage)*	Larvae palps	L3 larvae (early stage)	L3e
L2 larvae (mid-stage)	Adult proboscis	L3 larvae (late stage)	L3l
L2 larvae (late stage)*	Adult brains	L6 larvae (late stage)	L6l
L3 larvae (early stage)*	Larvae heads	Dimboa treated midguts§	MD
L3 larvae (mid-stage)	Hemocytes and imaginal discs	L4 and L5 larvae antennae and palps§	AP
L3 larvae (late stage)*	Salivary glands	Induced fat body§	Fbi
L6 larvae (early stage)	Gonads from female pupae	L5 larvae tracheae§	TrL6
L6 larvae (mid-stage)	Gonads from male pupae		
L6 larvae (late stage)*	L5&L6 larvae tracheae		
Male pupae	Gut stem cells		
Female pupae			

This Table shows the description of the samples from which RNA has been extracted. Columns A and B show samples sequenced by 454. They consist of 14 whole organisms developmental time-points (clumn A) and 13 dissected tissues (column B). Column C shows 10 samples sequenced by Illumina and column D shows the abbreviation of the Illumina libraries names that are used in the figures of the paper.

*Those six samples in column A, corresponding to whole organisms developmental time-points, have also been sequenced by Illumina (Column C).

§Those four samples in column C, correspond to dissected tissues that have been sequenced by Illumina only and that were not included in the pooled sample for 454 sequencing.

**Table 2:** **Statistics of the Sf_TR2012b transcriptome assembly compared to** ***D. melanogaster*** **and** ***B. mori***

	Sf_GATC_Clusters	Sf_TR2012b	Dm_transcripts_r5.50	Bm_assembled_ESTs
Sequence numbers	183,373	54,976	28,538	16,425
Total length (nt)	61,002,208	36,925,829	81,145,340	11,205,779
N50 (nt)	408	876	3,856	676
N90 (nt)	295	400	1,447	475

The data for *D. melanogaster* has been downloaded from Flybase (ftp://ftp.flybase.net/genomes/Drosophila_melanogaster/dmel_r5.50_FB2013_02/fasta/dmel-all-transcript-r5.50.fasta.gz) while the data from B. mori has been downloaded from SilkDB (ftp://ftp.genomics.org.cn/pub/SilkDB/cDNA/Silkworm_unigenes/SW_unigene.seq). In *D. melanogaster*, all transcripts correspond to the latest version of transcript annotation coming from gene prediction and manual curation. *B. mori* dataset has been generated by assembling a collection of ~64,000 ESTs, giving two reference points for different transcriptomic strategies.

### Evaluation of the Sf_TR2012b assembly

**Figure 1:**
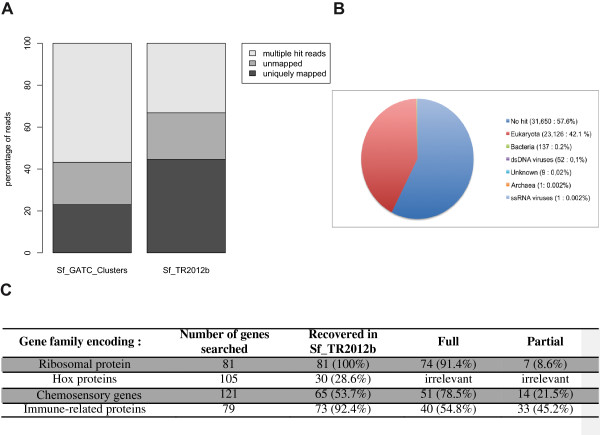
**Content of the reference transcriptome. A**. Barplot representing the percentages of multiple hit reads, unmapped reads and uniquely mapped reads, as provided by Bowtie, when aligning an RNAseq library against either the Sf_454_clusters assembly or the Sf_TR2012b assembly. The percentages obtained are the average of four independent experiments. **B**. Pie chart representing the number and percentage of contigs from Sf_TR2012b grouped by their best blastx hit against nr. Number of contigs and percentage of the total are represented. **C**. Synthetic table representing the number of genes found per family in the Sf_TR2012b assembly. The number of full and partial transcripts for Hox-domain proteins is irrelevant because the only conserved part is the homeodomain itself.

First, we wanted to evaluate whether the last step of assembly, combining the short reads with the long reads sequences, was improving the quality of our transcriptome. We evaluated this improvement by aligning independent Illumina libraries on the different reference transcriptome assemblies using the Bowtie software [7]. We used 4 .fastq files containing sequences for whole larvae RNA extracted in experimental conditions, which were unrelated to our transcriptome project (N. Volkoff, personal communication), to align the reads against Sf_TR2012b and Sf_GATC_clusters. For all 4 samples, the average of unmapped reads is around 20% when aligned on both assemblies (Sf_GATC_Clusters and Sf_TR2012b) (Figure 1A). However we greatly improved the percentage of uniquely mapped reads from 22% to 45% while decreasing the number of multiple reads from 58% to 32% (Figure 1A). This indicates that our second step of assembly was particularly effective at eliminating most of the redundancy that one could expect from an organism that is far from being isogenic.

Then, we wanted compare our approach with other insects datasets for which the transcriptome was obtained differently. We thus compared the number of contigs (55,000) and the total size (37 M nt) of the Sf_TR2012b assembly with 2 other insect models, *Drosophila melanogaster* and *Bombyx mori*. The *Drosophila melanogaster* transcriptome contains more than 28,000 sequences for a total length of 81 M nt (Table 2). The Drosophila transcripts set is built primarily from computer predictions of the genome sequence and also from permanent curations due to the large Drosophila research community and more than a hundred years of genetics research on this model. Due to the conservation of the majority of genes between insects, we should expect a “complete” transcriptome to come close to this number. A standard approach to construct a reference transcriptome is Sanger sequencing of an EST library. We can find an example of this in public repositories for *Bombyx mori*. We can see that the total size of this assembly is around 11 Mb (Table 2) because of the assembly of Sanger sequences in Unigenes.Then, we seeked to have a better view of the total content of the Sf_TR2012b assembly by performing systematic blastx against nr (Figure 1B) to checked whether our RNA sequences were corresponding to bona fide proteins and also to checked whether we had some contaminants. 23,126 (42.1%) of the contigs were matching eukaryotic proteins. Of those, 19,895 (86.0%) contigs are similar to a Heliconius protein (Hmel1-1_Release_20120601), while 9,709 Heliconius proteins (75.7%) are similar to a TR2012b contig.

Similarly, 21,439 (92.7%) contigs are similar to a Monarch protein (Dp_geneset_OGS2), while 10,887 Danaus Plexippus (72.0%) proteins are similar to a TR2012b contig. Both comparisons have been made using blastx with a threshold of 1e-10 (p-value) without complexity filter.

We found only 200 contigs (0.4%) matched prokaryotes (archea, bacteria and viruses), making large scale contaminations of our samples highly improbable. What was more surprising was that 57.6% of our RNA sequences didn’t match any known protein sequences. Among these 31,650 sequences without hit against the NR databank, 491 include at least one protein domain found with the following algorithms (BlastProDom, FPrintScan, Gene3D, HMMPanther, HMMPfam, HMMSmart, PatternScan, ProfileScan and superfamily). These comparisons have been done using Interproscan (v4.8) directly on the transcript sequences. This low number of hit does not necessarily reflect that the other sequences are spurious, but they may derived from UTR, or the predicted ORF (Interproscan uses getorf from emboss) might be uncompleted or the sequences may contain a frameshift. This is enhanced by the observation that the transcripts without hit are shorter than the transcripts with hits (mean : 526.5, median : 446 versus global mean : 868.7, and global median : 694). Finally, some of the transcripts without any match against NR databank might correspond to lncRNA, as well as transcribed repeat elements.

For the sequences matching eukaryota, we performed blastx against eukaryotic core gene sets such as the CEGMA (v2.5) geneset (http://korflab.ucdavis.edu/datasets/cegma/) and the BUSCO proteins set (ftp://cegg.unige.ch/OrthoDB7/BUSCO). We found that 452 among 457 CEGMA proteins (98.9%) are similar to 1831 Sf_TR2012b contigs. Similarly 2961 among 3369 BUSCO drosophila proteins (87.9%) and 3025 among the 3299 BUSCO *Danaus plexippus* proteins (91.7%) were present in the Sf_TR2012b assembly, suggesting that the core components of the FAW transcriptome were present in our assembly.

### Assessment of Sf_TR2012b quality and usability through gene families annotation

In order to evaluate the proportion of genes present/absent from our reference transcriptome, we manually annotated different families of genes. Ribosomal proteins (rbp) are mostly conserved among eukaryota and are present as highly expressed small genes throughout most genomes. Thus the percentage of rbp found should be indicative of the minimal requirement for finding most housekeeping genes. We used a set of 81 proteins annotated as rbp in *B. mori* (D. Heckel, personal communication) (Additional file 3: Table S2) and searched for homologs in Sf_TR2012b. Out of those 81 sequences, we could find 74 hits matching the complete *B. mori* transcript. For 7 rbp, we found only partial matches (Figure 1C). Thus, we conclude that more than 90% of housekeeping genes are represented in Sf_TR2012b assembly.

Conversely, we searched for more constrained genes belonging to 3 functional families : homeobox-domain genes (Hox), odorant and pheromone-binding proteins and immune related genes. Hox proteins can be identified with certainty thanks to the conserved signature of their homeodomains even between distantly related species [8]. Within species however, many paralogs can be identified. Contrary to the ribosomal proteins, the expression of genes encoding Hox proteins in *D. melanogaster* is usually temporally and spatially restricted. Thus we expected these particular proteins to be more difficult to find in our assembled transcriptome due to their underrepresentation in the RNA samples collected, compared to other abundant transcripts such as ribosomal proteins. We used a collection of 105 *D. melanogaster* homeodomain protein sequences from the Homeodomain Resource Database [9] as a tblastn query of our Sf_TR2012b transcriptome assembly. 30 (28.6%) unique *Drosophila* homeodomain orthologs were thus detected (Additional file 4: Table S3, Figure 1C).

Similarly, we used a set of chemosensory genes previously identified by transcriptome sequencing in the closely related species *S. littoralis*, the cotton leaf worm [10–12], and that comprises both highly expressed (odorant-binding proteins, OBPs, and chemosensory proteins, CSPs) and low expressed (chemosensory receptors) genes. Found in abundance in the olfactory organs, OBPs and CSPs are proposed to transport odorants to membrane bound receptors [13, 14]. Two families of volatile molecule receptors have been described in insects, the olfactory receptors (ORs) and the ionotropic receptors (IRs), these two types being involved in the recognition of different volatile families as demonstrated in *D. melanogaster* [15]. Co-receptors highly conserved among species are required for these receptor functioning: ORco [16–18] is required to form with ORs heterodimers while IR25a and IR8a are proposed to complex with IRs [19]. We used a set of 121 chemosensory genes (36 OBPs, 21 CSPs, 47 ORs and 17 IRs) previously identified in *S. littoralis* to search for homologs in the *S. frugiperda* reference transcriptome. 50 (87.7%) of the highly expressed transcripts (OBPs and CSPs) were recovered whereas we could recover only 15 out of 64 *S. littoralis* low expressed chemosensory receptors transcripts (23.4%) (Figure 1C). Interestingly, we could identify 11 putative new chemosensory transcripts, bearing the hallmark signal peptide but with no ortholog in S. littoralis, encompassing 7 OBPs, 3 CSPs and one IR). The numbers of OBPs and CSPs annotated in *S. frugiperda*, (38 OBPs and 22 CSPs - Additional file 5: Table S4) is within the range of the numbers of OBPs and CSPs usually annotated in Lepidoptera genomes, e.g. in *B. mori* [20, 21]. Only partial sets of ORs and IRs could be identified compared to the numbers of such genes annotated in either *S. littoralis* or *B. mori* [22, 23], certainly because of their low expression level. Accordingly, we found many frameshifts and inappropriate stop codons in the predicted ORs. However, the three co-receptors ORco, IR25a and IR8a could all be annotated in *S. frugiperda.* This reflects their high expression levels due to their function as co-receptors.

**Figure 2:**
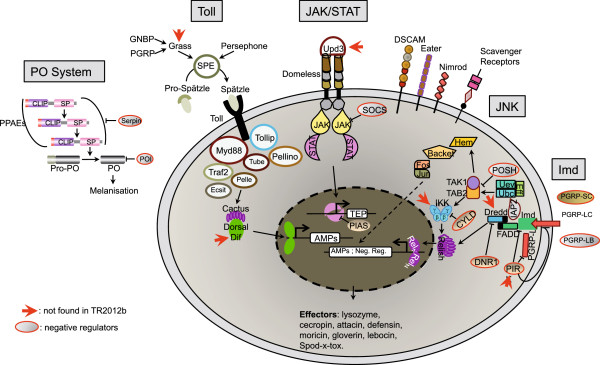
**Schematic representation of** ***S. frugiperda*** **immune components found in Sf_TR2012b.** The four main signaling pathways involved in insect immune response are detailed as well as the pro-PO cascade. The negative regulators are red circled and the components that were not found in Sf_TR2012b are indicated by red arrows.

Finally, we tried to annotate most of the immune-related genes of FAW using Sf_TR2012b. The invertebrate immune response has been extensively studied in insects and today it is in the insect model, *D. melanogaster*, that we have the most integrated understanding of this physiological function. Indeed, biochemical, genetic and molecular biology approaches have led to the characterization of the molecular mechanisms involved in (i) pathogen recognition and extra-cellular signaling, (ii) signal transduction through intra-cellular signaling pathways, and (iii) pathogen elimination through the production of effectors molecules and cell activation (for review see [24, 25]). We inventoried the components of *S. frugiperda* immune repertoire by comparing Sf_TR2012b with the immune repertoire described for *D. melanogaster* and other insects [26–30] and classified them in three groups (Additional file 6: Table S5). The first one contains transcripts encoding proteins involved in pathogen recognition as well as extracellular molecules associated to signal transduction. The second group contains proteins belonging to intra cellular signaling pathways which control among others the antifungal, antibacterial and antiviral responses and that also play a key role in developmental processes (Toll, Imd, JAK/STAT and JNK). The third group contains an inventory of effectors of the immune response (mainly anti-microbial peptides, AMPs). As summarized in Figure 2, we were able to identify in *S. frugiperda* transcriptome most of the components involved in the *Drosophila* immune response (73 out of 79). Two of the missing components were located on the Toll pathway. The first one, Grass is a serine protease involved in the activation of spätzle processing enzyme. Grass belongs to a large family of CLIP domain containing proteases. In Sf_TR2012b, we identified 16 such proteases while 15 and 37 were found in the genomes of *B. mori* and *D. melanogaster*, respectively. Therefore, even though Grass might be one of them, we were not able to identify it with certainty. The second one is the Dorsal-related immunity factor, Dif, which, to our knowledge, was characterized only in *Diptera*. Three components of the Imd pathway, the inhibitor of kinase kinase gamma also known as Kenny in *Drosophila*, the caspase Dredd and the negative regulatory factor PIRK were not found. The last missing component is the cytokine Upd3, an activator of the JAK/STAT pathway which was also characterized only in Diptera.

Thanks to these four points of comparison, we think on one hand our current assembly is sufficiently deep to uncover most genes of *S. frugiperda*. They are usually complete sequences if they have a high level of expression. But on the other hand, we might have missed around 70% of the rarest transcripts. Altogether, the Sf_TR2012b assembly seems perfectly adequate in order to identify a large part of the coding sequences of *S. frugiperda*.

### Access to Sf_TR2012b through Lepidodb

**Figure 3:**
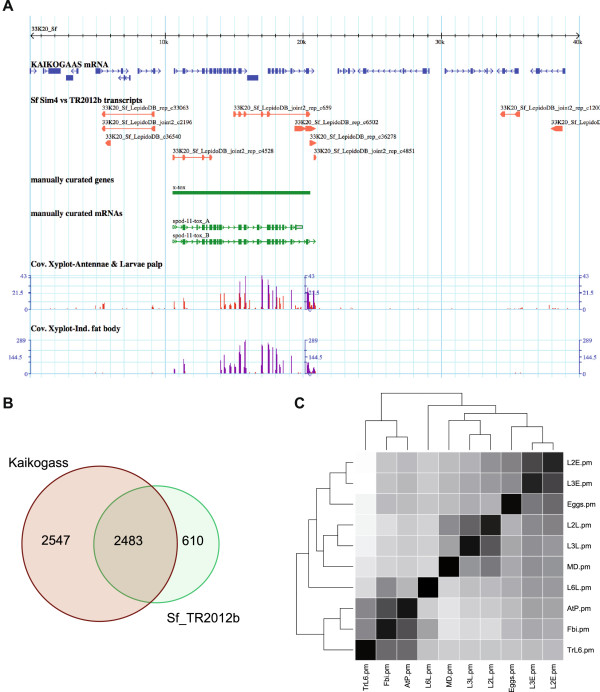
**Representation of transcripts and their expression. A**. Screenshot of the GBrowse system integrated in the Lepidodb. A region centered around spod-11-tox [34] is represented on the 33K20_Sf BAC. The manual annotation of this already described gene can be compared with KAIKOGASS_mRNA predictions (top track) and Sf_TR2012b transcript (second to top track). The bottom two tracks represent coverage reads from 2 Illumina tissue RNAseq experiments, induced fat bodies and larval antennae and palps. **B**. Venn diagram showing the overlap between Kaikogass gene predictions on the BACS and Sf_TR2012b transcripts aligned on the same BACs. **C**. Correlogram of the rpm values for each of the developmental time points and tissue RNAseq experiments.

The Sf_TR2012b assembly sequences can be downloaded through the Lepidodb database (http://www6.inra.fr/lepidodb/Private and http://www.inra.fr/lepidodb/downloads/TR2012b) [login: lepiduser password: papillon]. In this database, the transcripts can be queried with their identifier or with the name of an ortholog. BLASTx against the nr database have been performed for all transcripts in the assembly and the database contain the best 10 results for each transcript. BLAST searches can also be performed from the database. In addition a GBrowse system has been added that presents a set 44 BAC sequences (25 BAC sequences have been added in LepidoDB to the 19 already published [31]) representing each around 100 kb of contiguous genomic sequence, on which a computed gene prediction has been performed by KAIKOGAAS (http://kaikogaas.dna.affrc.go.jp/). We aligned the Sf_TR2012b transcripts on this genomic reference [31]. The alignment results by blast have been converted into a .gff3 file directly viewable in a GBrowse genomic viewer instance [32] hosted through the Lepidodb portal (http://www6.inra.fr/lepidodb/Private) (Figure 3A). Throughout the BACs, we have 49.4% of KAIKOGAAS gene predictions that overlap with an Sf_TR2012b transcript, while 80.3% of Sf_TR2012b overlap with a gene prediction (Figure 3B). We also provide for each transcript its level of expression for 10 different samples. These levels of expression are also represented on the BACs present in LepidoDB as tracks (Figure 3A) [33].

### Measure of transcripts expression by RNAseq

**Figure 4:**
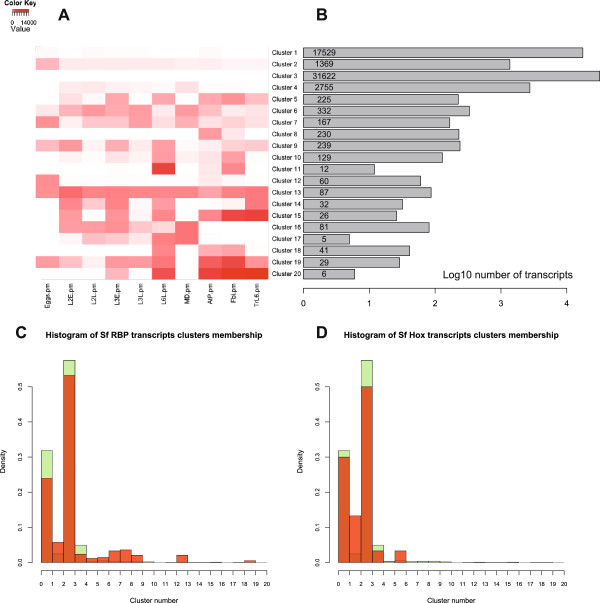
**Clustering of expression. A**. Heatmap representing the medoids of expression of the 20 clusters of genes for the 10 Illumina RNAseq experiments. **B**. Barplot representing the number of genes that are present in each cluster. **C**. Histogram representing the density of genes in each cluster for all (green) or RBP genes (red). Orange represents the intersection. It shows if RBP genes are over- or under-represented in each cluster compared to the total number of genes in each cluster. **D**. Same as in **C**. for Hox-domain genes.

We were interested in using the reference transcriptome to study families of genes involved in larval development, in chemosensory reception and in immune response. These categories of genes are all necessary to the adaptation of the feeding larva to its environment, whether be its host plant or its panoply of pathogens. Specifically we were interested in investigating the repertoire of genes and their level of expression for each of these categories. Thus, we extracted total RNA corresponding to 6 developmental time-points and 4 experimental dissected tissues to generate a quantitative gene expression dataset by Illumina sequencing (see Methods and Table 1). Total RNA has been extracted from the selected samples and sequenced to produce 10 M single-end 50 bp reads (see Additional file 7: Figure S2), representing approximately 1.3× coverage of each nucleotide in the reference transcriptome. Levels of expression for each gene in Sf_TR2012b have been measured by 4 indices: total coverage, reads per million (RPM), normalized reads count using limma [35] or normalization of reads count using DESeq [36]. These four tables of expression are available for download on the homepage of Lepidodb (http://www.inra.fr/lepidodb/downloads/TR2012b). We calculated the correlation coefficient (Pearson r) between pairwise samples (Figure 3C) and noted that there was much more correlation between L2 early and L3 early stages and between L2 late and L3 late than between both L2 stages or between both L3 stages, indicating that different transcripts expression profiles were defining the beginning and the end of each ecdysis cycle. Interestingly, the L6 late time-point was not correlated with the other larval time-points, probably reflecting the onset of metamorphosis. Less variation was observed between tracheae, antennae and palps and fat body samples, all coming from late larval stages.To identify the genes specific to each of the samples, we used a k-mean clustering method (using the clara function in R), to group together genes with similar expression profiles. We empirically chose 20 as the number of k-mean clusters that was producing categories with enough differences between them. We immediately noticed that one category (cluster 3, see Figure 4A-B) comprised most of the transcripts (31,622). This category had a mean expression level of close to 0 RPM. This probably represents most of the rare transcripts coming from the large amount of samples that we dissected and included in our 454 sequencing assembly, in addition to the set of ESTs previously described. The Cluster 1, similarly, had a large number (17,529) of transcripts with low expression and no noticeable specific expression profile. However, most of the other clusters identified groups of genes whose expression was specific of one or two samples (Ex, clusters 2 and 19 on Figure 4A). To test whether those clusters made sense, we checked to which clusters rbp and Hox proteins belonged. Compared to the overall distribution of transcripts in the different clusters, rbp proteins are enriched in clusters 2,5,6,7,8,9,13 and 19 (Figure 4C). Except for clusters 2 and 8, all the other clusters show a median level of expression that is high in all samples, even if this level varies from sample to sample. Hox transcripts however are enriched in clusters 2 and 6 (Figure 4D). The cluster 2 in particular corresponds to genes that have a higher level of expression during embryonic stages compared to any of the other samples, which makes sense given that Hox-domain genes are often transcription factors involved in embryogenesis.

### Common and specific developmental genes

**Figure 5:**
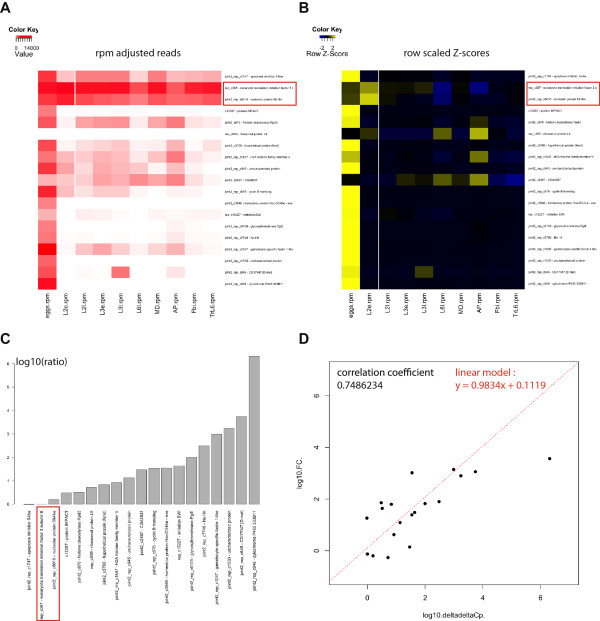
**qPCR validation of differential expression. A**. Heatmap representing the expression in rpm of each candidate transcript tested by qPCR. Genes are ordered from top to bottom from a lesser ratio between eggs and L2e samples, as measured in qPCR to a higher ratio. The red box shows the 2 genes that we used to normalize the qPCR. **B**. Same heatmap as in A. but showing expression as z-scores scaled by row to highlight the differential expression between eggs and L2e. **C**. Barplot showing the ratio measured in qPCR, using elongation factor 3 as a negative control for normalization. **D**. Scatter plot showing the correlation between fold changes measured by RNAseq (y axis) and ratios measured by qPCR for the tested genes. The two measurements have a correlation coefficient of 0.74. A linear regression model has been applied and is also shown on the same graph.

When designing the reference transcriptome, we emphasized the production of 6 developmental time-points specific RNA libraries to be sequenced by Illumina. We were interested in the specificity of development of *S. frugiperda* compared to other insects. Indeed, *S. frugiperda* is a pest in its larval stage and resistance to common pesticides has become a particularly prevalent issue [37]. We reasoned that identifying developmental genes specific to *S. frugiperda* would provide the community with specific targets for the development of new strategies of pest control. To identify genes involved in the embryonic development of *S. frugiperda*, we focused on the comparison of 2 Illumina RNAseq libraries sequenced from eggs and L2 stage larvae RNA extracts. We used the R framework package DEseq to identify genes that are overexpressed in eggs and no longer in L2 and that are thus required only during embryonic development. 117 genes have been identified with a striking pattern of embryo only expression (Additional file 8: Figure S3). We randomly selected 16 candidates from this list to confirm by quantitative PCR that our candidates correspond to genes that are effectively transcribed in *S. frugiperda* and are effectively transcribed in a regulatory fashion (Figure 5A-B). We also chose 2 negative controls with high expression at all stages (elf3 and nucleolar protein 58-like) and an rbp protein (rbpL8) with non detectable expression at all stages. 15/16 tested had a significantly higher expression in embryos than in L2 larvae in qPCR (Figure 5C). In addition, we observed that our differential expression measurements by RNAseq and by qPCR are linearly correlated (Figure 5D). While some of the genes we selected are well known in other organisms to regulate development (such as even-skipped, rpd3 or ISWI), we also included genes for which no clear orthology was detected. These transcripts, such as joint2_rep_c945, joint2_rep_c7748 and joint2_rep_c1530 might represent Lepidoptera specific embryonic genes important for embryonic development, which makes them interesting targets for the development of new pest control strategies.

### Expression of the genes of olfaction

**Figure 6:**
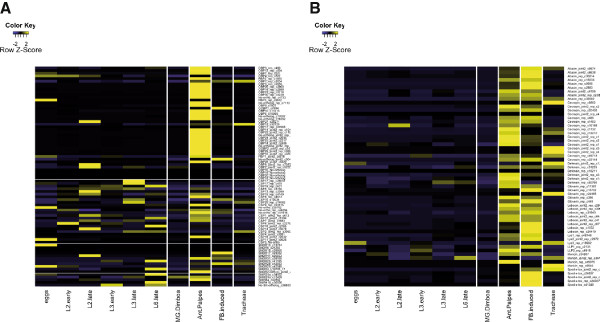
**Tissue specific expression of chemosensory genes and anti-microbial peptides. A**. Heatmap showing the expression as row scaled z-scores of *S. frugiperda* odorant-binding proteins, chemosensory proteins, olfactory receptors and ionotropic receptors in the 10 Illumina RNAseq experiments. A higher expression of odorant-binding proteins in the antennae and palps can be observed. **B**. Same as in **A**. for the anti-microbial peptides identified in *S. frugiperda* transcriptome. A higher expression of AMP in induced fat bodies is observed as well as an overexpression in antennae and palps as well as in tracheae, two tissues in contact with the external environment.

Both antennae and maxillary palps house olfactory neurons that detect odorant volatile molecules via different steps, each involving a specific family of proteins. As expected for proteins involved in olfaction, most of the OBPs and CSPs are highly expressed in antennae and palps (Figure 6A). Interestingly, while most of the OBPs expression was not visible when entire animals were used for RNA extraction (transcript “dilution”), many CSP transcripts could still be highly visible in different samples, suggesting that their expression is not restricted to the olfactory organs. This correlates well with some evidences that CSP function would not be restricted to olfaction [38, 39] and that these proteins would act in fact as general carriers of hydrophobic molecules throughout the insect body. Interestingly, the expression of some ORs was not restricted to the olfactory organs and several could be observed as expressed in the fat body or the midgut (Figure 6A). Such ectopic expression has been already described for some ORs in insects, for instance in *B. mori* abdmonen [40] and gut [22] and in *S. littoralis* abdomen and brain [10, 11], although the function of ORs in such organs is still unknown. IR25a showed a broad expression, as already observed in *S. littoralis* [41].

### Expression of the genes of immunity

Analyses of immune genes expression in the different development stages or tissues are in agreement with what is commonly described in the literature. For instance, induced fat body (FB) strongly expressed genes encoding AMPs (Additional file 9: Figure S4, Figure 6B). On the other hand, surprisingly, antennae and palps (A/P) also strongly expressed genes encoding AMPs. Interestingly, some AMPs genes are expressed in FB and A/P whereas others seem to be preferentially expressed in only one of the two “tissues”. What is the most remarkable is that some AMPs genes (i. e., some cecropins and defensins) are less expressed in bacterial challenged FB than in unchallenged A/P, suggesting that those tissues develop a constitutive immune response.

Thanks to high-throughput sequencing of antennal transcriptomes, this phenomenon has recently been observed in other Lepidoptera, in Diptera (reviewed in [42]) and in some Hymenoptera like the leaf-cutter ant in which most of the immune genes are highly expressed in the antenna of the queen ant [43]. Those observations raise the question of the interplay between immune and gustatory/olfactory systems. One may wonder if expression of immune genes in gustatory/olfactory systems is a bona fide immune response or is somehow involved in nutritional strategies like food selection.

## Conclusions

In conclusion, we provide in this study a reference transcriptome for *S. frugiperda*, available through a dedicated database, along with measures of differential expression across 10 different samples. We found this resource invaluable to annotate and study the expression of different families of genes. In particular, we were able to identify and validate Lepidoptera specific genes involved in development. We also analyzed a set of genes involved in olfaction across two *Spodoptera* species. And finally, we annotated an almost complete set of immune related genes and observed in particular that some anti-microbial peptides are highly expressed in chemosensory organs, even in absence of induction, raising the possibility that antennae and palps can naturally act as primary organs of immune response, since they are in open contact with the natural environment.

## Methods

### RNA extraction

Total RNA has been extracted using Trizol® reagent according to manufacturer recommendations from the biological samples indicated in Table 1. For each condition, whether a developmental time point or a dissected tissue, a sufficient amount of fresh tissue has been used in order to extract around 10 μg of total RNA. Staging of the larval time-points were made according to the size of the cephalic capsule combined with the time elapsed from one stage to another. Dissections of were performed in standard conditions without any peculiarities except for gut stem cells isolation, where midgut tissues were dissected from anesthetized larvae just before the 5th molt and stem cells were isolated as previously reported [44].

2 samples in particular were subjected to specific conditions.

1. the FatBody induced sample, (library Fbi from Table 1, column C) 1 day-old *S. frugiperda* sixth-instar larvae were bacteria-challenged with a mixture of *Escherichia coli* (CIP7624) and *Micrococcus luteus* (CIP5345) (10^6^ bacteria/larva). Eight hours post infection, the fat body from 6 larvae was recovered and RNA extracted.
2. the Midgut DIMBOA sample (MD library, Table 1 column C), L5 larvae were fed corn plants of the Ci31A variety that contains high levels of DIMBOA. DIMBOA (2,4-dihydroxy-7-methoxy-1,4-benzoxazin-3-one) is an antibiotic molecule naturally present in maize that protects it from pests and pathogens. Midguts were then dissected and washed prior to RNA extraction.

For the 454 sequencing, a normalized pool (equimolar for each sample from the Table 1 columns A and B) has been prepared. 37 μg of RNA from the pool has been used for the construction of the library. 10 μg of RNA has been extracted for each of the10 samples destined to Illumina sequencing (Table 1, column C).

### Sequencing and statistics

The RNA samples have been sent to the GATC Company for 454 sequencing (construction of one normalized cDNA library and sequencing on the GS FLX (Roche/454), Titanium chemistry) and Illumina sequencing (construction of tagged standard cDNA libraries and sequencing of 1 × 56 bp on a Genome Analyzer II (Illumina/Solexa)) according to manufacturers instructions. The 454 sequencing generated 1,080,352 reads of a mean length of 322 bp. The number of reads generated by Illumina for each library are indicated in Additional file 7: Figure S2 and are in the range of 3 to 11 millions reads.

### Assembly and alignments

The flowchart of the assembly process is presented in Additional file 1: Figure S1. A first step of assembly of the 454 reads has been performed by GATC Company using the CD-HIT software [45] and resulted in 183,373 clusters. The 1,042,944 454 reads clipped from adaptator (GATC) were compared to Univec (https://www.ncbi.nlm.nih.gov/tools/vecscreen/univec/, version of march 2011) leading to the removal of 3,297 reads similar to known vector sequences. The 79,148 ESTs previously sequenced by Sanger method and coming from 8 different libraries (Sf9 cell lines, Sf21 cell lines, hemocytes, induced hemocytes, midgut, induced midgut, fat body and a tissue mix) [6] were also compared to UniVec resulting in the removal of 1132 ESTs. We performed an assembly of the 78,016 ESTs and 1,039,647 454 reads using the MIRA software [4]. This step resulted in 52,865 contigs with an N50 of 794 bp. Then we clipped the sequences at both ends by 80 bp and mapped the combined 90,454,901 reads from the 10 Illumina libraries onto this reference with Gassst [46]. The resulting 17,724,510 short reads that were unmapped underwent a subsequent step of short reads assembly using the velvet and Oases softwares [47]. We finally used MIRA to assemble together the 24,505 contigs from the Velvet/Oases assembly and the 52,865 contigs from the previous MIRA assembly of the 454 sequences and EST sequences. This final assembly is Sf_TR2012b.

To produce the time-course expression datasets, the reads from the 10 Illumina libraries have been aligned against Sf_TR2012b and the BACs set using bowtie [7]. Alignment files were further processed by samtools [48].

We compared the Sf_TR2012b proteins to the *Bombyx mori* GeneSet A B and C (http://sgp.dna.affrc.go.jp/ComprehensiveGeneSet/) using blastx with a threshold of 1e-10 (p-value) without complexity filter.

### Analysis of expression data

The Illumina 50 bp reads were mapped on the transcriptome with bowtie using the options (-a -m 1 --best --strata), reporting the best alignment (i.e. having the least number of mismatches). The raw counts by library were calculated by contig using a home-made program (based on the perl Bio::DB::Sam library). The raw counts have been divided by the total number of aligned reads in order to obtain the RPM normalized values. As well, for each library we computed the normalizations by quantile normalize BetweenArrays function of the limma R library [32] and the by size factors (functions estimateSizeFactors and sizeFactors from the DESeq R package [33]).

### BACs

25 BACs from *Spodoptera frugiperda* genome have been isolated as previously described [31]. Their sequence has been deposited at the European Bioinformatics Institute nucleotide archive : http://www.ebi.ac.uk/ena/. The accession numbers are listed in Additional file 10.

### qPCR

For the qPCR validation we extracted RNA from independent samples of *Spodoptera frugiperda* eggs and L2 larvae. A reverse transcription has been performed, using SuperscriptIII from Invitrogen to obtain the cDNA. We performed SYBRGreen (Roche) based qPCRs on 384-well plates on a LightCycler 480. Each reaction has been performed in triplicate on the plate. The quantification method was ∆∆Cp. In addition, we performed the qPCR validations for all the primers on 3 independent biological replicates for eggs and L2 larvae. Primers sequences are indicated in the Additional file 10.

## Availability of supporting data

All datasets are publically available through the LepidoDB interface at http://www6.inra.fr/lepidodb/Downloads. Additionally BAC sequences are available at http://www.ebi.ac.uk/ena/. The accession numbers are listed in Additional file 10.

## Electronic supplementary material

Additional file 1: Figure S1: Flowchart describing the Sf_TR2012b assembly process. (PDF 1 MB)

Additional file 2: Table S1: Statistics of the Sf_TR2012b assembly. (PDF 158 KB)

Additional file 3: Table S2: Ribosomal orthologous transcripts present in the Sf_TR2012b assembly. We used a manual annotation of *Bombyx mori* ribosomal proteins (David Heckel, personal communication) to search the transcripts present in our assembly using the tblastn algorithm [49, 50]. The ribosomal protein names in the first column follow the nomenclature developed for the rat. The one exception is RpS11 that exists as two closely related paralogs in all Lepidoptera (even *Drosophila*). However, both BmRpS11-1 and BmRpS11-2 match with the same transcript in our transcriptome assembly: joint2_rep_c105. In this table, we present every match that has over 50% identity and over 50% coverage of the query sequence. blastx against nr have been performed and the best hit for each S. frugiperda transcript has been represented with its evalue. In the last column, we dubbed a *S. frugiperda* sequence full if it has over 80% coverage and over 80% aminoacids identity with the query sequence (first column). It is ‘partial’ if it has less than 80% coverage. (PDF 41 KB)

Additional file 4: Table S3: Homeodomain protein orthologs in Sf_TR2012b. This table shows the tblastn results using 108 *Drosophila* homeodomain sequences from the Homeodomain Resource Database [9]. The legend is as in Additional file 3: Table S2 except that only the best Sf_TR2012b match for each Hox-domain sequence has been reported and coverage value have been omitted since we searched for a small domain only. For each of the *S. frugiperda* transcripts detected, a blastx of the transcript sequence against the nr database has been performed and the best hit has been reported in the table as well as the e-value of the hit. (PDF 57 KB)

Additional file 5: Table S4: Genes of olfaction. Candidate odorant-binding protein (OBP) and chemosensory protein (CSP) encoding genes identified in Sf_TR2012b by tblastn using 57 proteins annotated in the closely related species *S. littoralis* [10–12]. The occurrence of a signal peptide, a hallmark for OBPs and CSPs, is indicated (Y, yes; N, no). Full-length coding sequences are also indicated. Candidate olfactory receptor (OR) and ionotropic receptor (IR) encoding genes identified in Sf_TR2012b by tblastn using 64 proteins annotated in the closely related species *S. littoralis* [10–12]. Identified frameshifts or unexpected stop codons in the coding sequences are indicated (Y, yes; N, no). (PDF 171 KB)

Additional file 6: Table S5: Genes of Immunity. Innate immunity-related actors identified during *S. frugiperda* transcriptome wild analysis were classified in six groups. The first one contains transcripts encoding proteins involved in pathogen recognition as well as extracellular molecules associated to signal transduction. The second group contains proteins belonging to the Toll pathway which control among others the antifungal response and that also play a key role in developmental processes. The third group gathers proteins belonging to the Imd cascade that is at the center of the response against the Gram-negative bacteria. The fourth group lists the JAK/STAT pathway members; this pathway was originally identified through its role in embryonic segmentation, later, it was shown that this pathway is also involved in the innate immunity and stress response. The fifth group contains members of the JNK pathway. Finally, the sixth group made an inventory of all the effectors of the immune response. (PDF 138 KB)

Additional file 7: Figure S2: Expression time-course. Barplot showing the total number of reads for the 10 Illumina libraries from Table 1, column C. (PDF 774 KB)

Additional file 8: Figure S3: Candidate genes overexpressed in eggs. Heatmap showing the rpm normalized reads count of genes having more than 200 reads in eggs and less than 20 reads in L2e stage. (PDF 110 KB)

Additional file 9: Figure S4: Expression of immunity genes. A - B. Heatmaps showing the expression as row scaled z-scores of *S. frugiperda* genes of immunity in the 10 Illumina RNAseq experiments from Table 1, column C. (PNG 60 KB)

Additional file 10: Contains accession numbers for the BACs and qPCR primer sequences. (DOCX 71 KB)

## References

[1] Wetterstrand KA: *DNA Sequencing Costs: Data from the NHGRI Genome Sequencing Program (GSP)*. Available at: http://www.genome.gov/sequencingcosts. Accessed [date of access]

[2] Capinera JL (2001). Handbook of Vegetable Pests. Vol. 1.

[3] Pogue MG (2002). A World Revision of the Genus Spodoptera Guenée : (Lepidoptera: Noctuidae).

[4] Chevreux B, Wetter T, Suhai S (1999). Genome Sequence Assembly Using Trace Signals and Additional Sequence Information. Computer Science and Biology: Proceedings of the German Conference on Bioinformatics (GCB) 99: 1999.

[5] Mortazavi A, Williams BA, McCue K, Schaeffer L, Wold B (2008). Mapping and quantifying mammalian transcriptomes by RNA-Seq. Nat Methods.

[6] Negre V, Hotelier T, Volkoff AN, Gimenez S, Cousserans F, Mita K, Sabau X, Rocher J, Lopez-Ferber M, d'Alencon E, Audant P, Sabourault C, Bidegainberry V, Hilliou F, Fournier P (2006). SPODOBASE: an EST database for the lepidopteran crop pest Spodoptera. BMC Bioinformatics.

[7] Langmead B, Trapnell C, Pop M, Salzberg SL (2009). Ultrafast and memory-efficient alignment of short DNA sequences to the human genome. Genome Biol.

[8] McGinnis W, Krumlauf R (1992). Homeobox genes and axial patterning. Cell.

[9] Moreland RT, Ryan JF, Pan C, Baxevanis AD (2009). The Homeodomain Resource: a comprehensive collection of sequence, structure, interaction, genomic and functional information on the homeodomain protein family. Database (Oxford).

[10] Jacquin-Joly E, Legeai F, Montagne N, Monsempes C, Francois MC, Poulain J, Gavory F, Walker WB, Hansson BS, Larsson MC (2012). Candidate chemosensory genes in female antennae of the noctuid moth Spodoptera littoralis. Int J Biol Sci.

[11] Legeai F, Malpel S, Montagne N, Monsempes C, Cousserans F, Merlin C, Francois MC, Maibeche-Coisne M, Gavory F, Poulain J, Jacquin-Joly E (2011). An expressed sequence tag collection from the male antennae of the Noctuid moth Spodoptera littoralis: a resource for olfactory and pheromone detection research. BMC Genomics.

[12] Poivet E, Gallot A, Montagne N, Glaser N, Legeai F, Jacquin-Joly E (2013). A comparison of the olfactory gene repertoires of adults and larvae in the noctuid moth Spodoptera littoralis. PLoS ONE.

[13] Leal WS (2013). Odorant reception in insects: roles of receptors, binding proteins, and degrading enzymes. Annu Rev Entomol.

[14] Pelosi P, Gallot A, Montagne N, Glaser N, Legeai F, Jacquin-Joly E (2006). Soluble proteins in insect chemical communication. Cell Mol Life Sci.

[15] Silbering AF, Rytz R, Grosjean Y, Abuin L, Ramdya P, Jefferis GS, Benton R (2011). Complementary function and integrated wiring of the evolutionarily distinct Drosophila olfactory subsystems. J Neurosci.

[16] Benton R, Sachse S, Michnick SW, Vosshall LB (2006). Atypical membrane topology and heteromeric function of Drosophila odorant receptors in vivo. PLoS Biol.

[17] Larsson MC, Domingos AI, Jones WD, Chiappe ME, Amrein H, Vosshall LB (2004). Or83b encodes a broadly expressed odorant receptor essential for Drosophila olfaction. Neuron.

[18] Vosshall LB, Hansson BS (2011). A unified nomenclature system for the insect olfactory coreceptor. Chem Senses.

[19] Abuin L, Bargeton B, Ulbrich MH, Isacoff EY, Kellenberger S, Benton R (2011). Functional architecture of olfactory ionotropic glutamate receptors. Neuron.

[20] Gong DP, Zhang HJ, Zhao P, Lin Y, Xia QY, Xiang ZH (2007). Identification and expression pattern of the chemosensory protein gene family in the silkworm, Bombyx mori. Insect Biochem Mol Biol.

[21] Gong DP, Zhang HJ, Zhao P, Xia QY, Xiang ZH (2009). The odorant binding protein gene family from the genome of silkworm, Bombyx mori. BMC Genomics.

[22] Tanaka K, Uda Y, Ono Y, Nakagawa T, Suwa M, Yamaoka R, Touhara K (2009). Highly selective tuning of a silkworm olfactory receptor to a key mulberry leaf volatile. Curr Biol.

[23] Croset V, Rytz R, Cummins SF, Budd A, Brawand D, Kaessmann H, Gibson TJ, Benton R (2010). Ancient protostome origin of chemosensory ionotropic glutamate receptors and the evolution of insect taste and olfaction. PLoS Genet.

[24] Ferrandon D, Imler JL, Hetru C, Hoffmann JA (2007). The Drosophila systemic immune response: sensing and signalling during bacterial and fungal infections. Nat Rev Immunol.

[25] Lemaitre B, Hoffmann J (2007). The host defense of Drosophila melanogaster. Annu Rev Immunol.

[26] Dostert C, Jouanguy E, Irving P, Troxler L, Galiana-Arnoux D, Hetru C, Hoffmann JA, Imler JL (2005). The Jak-STAT signaling pathway is required but not sufficient for the antiviral response of drosophila. Nat Immunol.

[27] Kleino A, Silverman N (2014). The Drosophila IMD pathway in the activation of the humoral immune response. Dev Comp Immunol.

[28] Tanaka H, Ishibashi J, Fujita K, Nakajima Y, Sagisaka A, Tomimoto K, Suzuki N, Yoshiyama M, Kaneko Y, Iwasaki T, Sunagawa T, Yamaji K, Asaoka A, Mita K, Yamakawa M (2008). A genome-wide analysis of genes and gene families involved in innate immunity of Bombyx mori. Insect Biochem Mol Biol.

[29] Valanne S, Kallio J, Kleino A, Ramet M (2012). Large-scale RNAi screens add both clarity and complexity to Drosophila NF-kappaB signaling. Dev Comp Immunol.

[30] Zou Z, Evans JD, Lu Z, Zhao P, Williams M, Sumathipala N, Hetru C, Hultmark D, Jiang H (2007). Comparative genomic analysis of the Tribolium immune system. Genome Biol.

[31] d’Alencon E, Sezutsu H, Legeai F, Permal E, Bernard-Samain S, Gimenez S, Gagneur C, Cousserans F, Shimomura M, Brun-Barale A, Flutre T, Couloux A, East P, Gordon K, Mita K, Quesneville H, Fournier P, Feyereisen R (2010). Extensive synteny conservation of holocentric chromosomes in Lepidoptera despite high rates of local genome rearrangements. Proc Natl Acad Sci U S A.

[32] Stein LD, Mungall C, Shu S, Caudy M, Mangone M, Day A, Nickerson E, Stajich JE, Harris TW, Arva A, Lewis S (2002). The generic genome browser: a building block for a model organism system database. Genome Res.

[33] Stanojcic S, Gimenez S, Permal E, Cousserans F, Quesneville H, Fournier P, d'Alencon E (2011). Correlation of LNCR rasiRNAs expression with heterochromatin formation during development of the holocentric insect Spodoptera frugiperda. PLoS ONE.

[34] d’Alencon E, Bierne N, Girard PA, Magdelenat G, Gimenez S, Seninet I, Escoubas JM (2013). Evolutionary history of x-tox genes in three lepidopteran species: origin, evolution of primary and secondary structure and alternative splicing, generating a repertoire of immune-related proteins. Insect Biochem Mol Biol.

[35] Smyth GK, Gentleman VCR, Dudoit S, Irizarry R, Huber W (2005). Limma: Linear Models for Microarray Data. Bioinformatics and Computational Biology Solutions using R and Bioconductor.

[36] Anders S, Huber W (2010). Differential expression analysis for sequence count data. Genome Biol.

[37] Storer NP, Kubiszak ME, Ed King J, Thompson GD, Santos AC (2012). Status of resistance to Bt maize in Spodoptera frugiperda: lessons from Puerto Rico. J Invertebr Pathol.

[38] Gonga L, Luoa Q, Rizwan-ul-Haqa M, Hua MY (2012). Cloning and characterization of three chemosensory proteins from Spodoptera exigua and effects of gene silencing on female survival and reproduction. Bull Entomol Res.

[39] Kitabayashi AN, Arai T, Kubo T, Natori S (1998). Molecular cloning of cDNA for p10, a novel protein that increases in the regenerating legs of Periplaneta americana (American cockroach). Insect Biochem Mol Biol.

[40] Wanner KW, Anderson AR, Trowell SC, Theilmann DA, Robertson HM, Newcomb RD (2007). Female-biased expression of odourant receptor genes in the adult antennae of the silkworm, Bombyx mori. Insect Mol Biol.

[41] Olivier V, Monsempes C, Francois MC, Poivet E, Jacquin-Joly E (2011). Candidate chemosensory ionotropic receptors in a Lepidoptera. Insect Mol Biol.

[42] Siaussat D, Chertemps T, Maïbèche-Coisne M (2013). Detoxication, stress and immune responses in insect antenna: new insights from transcriptomics. Insect Biochem Mol Biol.

[43] Koch SI, Groh K, Vogel H, Hansson BS, Kleineidam CJ, Grosse-Wilde E (2013). Caste-specific expression patterns of immune response and chemosensory related genes in the leaf-cutting ant, Atta vollenweideri. PLoS ONE.

[44] Wang Y, Gosselin Grenet AS, Castelli I, Cermenati G, Ravallec M, Fiandra L, Debaisieux S, Multeau C, Lautredou N, Dupressoir T, Li Y, Casartelli M, Ogliastro M (2013). Densovirus crosses the insect midgut by transcytosis and disturbs the epithelial barrier function. J Virol.

[45] Fu L, Niu B, Zhu Z, Wu S, Li W (2012). CD-HIT: accelerated for clustering the next-generation sequencing data. Bioinformatics.

[46] Rizk G, Lavenier D (2010). GASSST: global alignment short sequence search tool. Bioinformatics.

[47] Schulz MH, Zerbino DR, Vingron M, Birney E (2012). Oases: robust de novo RNA-seq assembly across the dynamic range of expression levels. Bioinformatics.

[48] Li H, Handsaker B, Wysoker A, Fennell T, Ruan J, Homer N, Marth G, Abecasis G, Durbin R, Genome Project Data Processing S (2009). The sequence alignment/map format and SAMtools. Bioinformatics.

[49] Altschul SF, Gish W, Miller W, Myers EW, Lipman DJ (1990). Basic local alignment search tool. J Mol Biol.

[50] Altschul SF, Madden TL, Schaffer AA, Zhang J, Zhang Z, Miller W, Lipman DJ (1997). Gapped BLAST and PSI-BLAST: a new generation of protein database search programs. Nucleic Acids Res.

