# Supplementary material for: Establishment and analysis of a reference transcriptome for Spodoptera frugiperda
Source: BMC Genomics. 2014 Aug 23;15(1):704. doi: 10.1186/1471-2164-15-704 (PMC4150953; doi:10.1186/1471-2164-15-704)
Supplement: Supplementary file 2 — Additional file 2: Table S1: Statistics of the Sf_TR2012b assembly. (PDF 158 KB) [file 12864_2014_6384_MOESM2_ESM.pdf]

Bombyx mori ribosomal protein	Sf_TR2012b ID	Size (nt)	% identity between homologs	Best Blastx	E-value	Status
BmRpP0	joint2_rep_c11014	1309	94.55	>gi|18253041|gb|AAL62465.1||60S acidic ribosomal protein P0 [Spodoptera frugiperda]	0.0	Full
	joint2_rep_c205	1329	94.18	>gi|18253041|gb|AAL62465.1||60S acidic ribosomal protein P0 [Spodoptera frugiperda]	0.0	
BmRpP1	joint2_rep_c502	932	86.61	>gi|18253043|gb|AAL62466.1||60S acidic ribosomal protein P1 [Spodoptera frugiperda]	8E-39	Full
BmRpP2	joint2_rep_c115	736	89.39	>gi|18253045|gb|AAL62467.1||60S acidic ribosomal protein P2 [Spodoptera frugiperda]	1E-35	Partial
	rep_c50995	501	89.23	>gi|18253045|gb|AAL62467.1||60S acidic ribosomal protein P2 [Spodoptera frugiperda]	4E-36	
	rep_c49417	614	87.69	>gi|18253045|gb|AAL62467.1||60S acidic ribosomal protein P2 [Spodoptera frugiperda]	6E-36	
	rep_c23996	613	87.50	>gi|18253045|gb|AAL62467.1||60S acidic ribosomal protein P2 [Spodoptera frugiperda]	2E-41	
BmRpL3	joint2_rep_c500	1354	98.75	>gi|18253047|gb|AAL62468.1||ribosomal protein L3 [Spodoptera frugiperda]	0.0	Full
	joint2_rep_c137	1420	98.75	>gi|18253047|gb|AAL62468.1||ribosomal protein L3 [Spodoptera frugiperda]	0.0	
	joint2_rep_c11295	1259	87.50	>gi|18253047|gb|AAL62468.1||ribosomal protein L3 [Spodoptera frugiperda]	0.0	
	rep_c50038	1570	98.48	>gi|18253047|gb|AAL62468.1||ribosomal protein L3 [Spodoptera frugiperda]	0.0	Partial
BmRpL4	joint2_rep_c147	1440	88.71	>gi|170280411|gb|ACB12079.1||ribosomal protein L4 [Heliothis virescens]	0.0	Full
	rep_c47256	764	91.93	>gi|348019681|gb|AEP43782.1||ribosomal protein L4 [Biston betularia]	6E-129	Partial
BmRpL5	rep_c48073	1045	94.98	>gi|268306434|gb|ACY95338.1||ribosomal protein L5 [Manduca sexta]	0.0	Full
	joint2_rep_c4510	1116	94.98	>gi|268306434|gb|ACY95338.1||ribosomal protein L5 [Manduca sexta]	0.0	
BmRpL6	rep_c50488	972	82.52	>gi|161015753|gb|ABX55884.1||ribosomal protein L6e [Spodoptera exigua]	8E-138	Full
	joint2_rep_c37	1146	82.52	>gi|161015753|gb|ABX55884.1||ribosomal protein L6e [Spodoptera exigua]	5E-136	
	rep_c51101	719	82.35	>gi|161015753|gb|ABX55884.1||ribosomal protein L6e [Spodoptera exigua]	6E-99	Partial
	joint2_rep_c3541	906	78.71	>gi|161015753|gb|ABX55884.1||ribosomal protein L6e [Spodoptera exigua]	7E-83	
BmRpL7	joint2_rep_c80	923	94.76	>gi|18253049|gb|AAL62469.1||ribosomal protein L7 [Spodoptera frugiperda]	5E-175	Full
BmRpL7A	joint2_rep_c8183	1008	94.98	>gi|161015755|gb|ABX55885.1||ribosomal protein L7A [Spodoptera exigua]	6E-144	Full
	rep_c44992	1032	93.15	>gi|161015755|gb|ABX55885.1||ribosomal protein L7A [Spodoptera exigua]	2E-135	
	joint2_rep_c258	1088	84.02	>gi|161015755|gb|ABX55885.1||ribosomal protein L7A [Spodoptera exigua]	4E-122	
BmRpL8	rep_c559	930	100.00	>gi|112983487|ref|NP_001037141.1||ribosomal protein L8 [Bombyx mori]	2E-173	Full
	rep_c50865	872	99.58	>gi|112983487|ref|NP_001037141.1||ribosomal protein L8 [Bombyx mori]	6E-173	
	rep_c49083	945	99.17	>gi|112983487|ref|NP_001037141.1||ribosomal protein L8 [Bombyx mori]	1E-171	
	joint2_rep_c11304	982	98.75	>gi|112983487|ref|NP_001037141.1||ribosomal protein L8 [Bombyx mori]	2E-160	
	rep_c49993	885	98.33	>gi|112983487|ref|NP_001037141.1||ribosomal protein L8 [Bombyx mori]	6E-168	
	rep_c51053	883	98.18	>gi|112983487|ref|NP_001037141.1||ribosomal protein L8 [Bombyx mori]	2E-153	
	rep_c50808	744	99.02	>gi|112983487|ref|NP_001037141.1||ribosomal protein L8 [Bombyx mori]	2E-142	Partial
	joint2_rep_c11274	892	99.46	>gi|112983487|ref|NP_001037141.1||ribosomal protein L8 [Bombyx mori]	8E-131	
BmRpL9	joint2_rep_c2126	704	95.79	>gi|21759393|sp|Q963B7.1|RL9_SPOFR|ribosomal protein L9 [Spodoptera frugiperda]	1E-127	Full
	joint2_rep_c182	899	95.79	>gi|21759393|sp|Q963B7.1|RL9_SPOFR|ribosomal protein L9 [Spodoptera frugiperda]	4E-126	
BmRpL10	joint2_rep_c1668	953	95.43	>gi|160947858|gb|ABX54738.1||ribosomal protein L10 [Spodoptera exigua]	6E-162	Full
	rep_c49619	794	94.98	>gi|160947858|gb|ABX54738.1||ribosomal protein L10 [Spodoptera exigua]	1E-161	
	joint2_rep_c23	871	94.06	>gi|160947858|gb|ABX54738.1||ribosomal protein L10 [Spodoptera exigua]	2E-159	
BmRpL10A	joint2_rep_c10979	779	97.24	>gi|22001886|sp|Q963B6.1|RL10A_SPOFR|ribosomal protein L10A [Spodoptera frugiperda]	6E-134	Full
	joint2_rep_c1822	787	97.24	>gi|22001886|sp|Q963B6.1|RL10A_SPOFR|ribosomal protein L10A [Spodoptera frugiperda]	3E-134	
	joint2_rep_c11054	804	93.98	>gi|342356339|gb|AEL28828.1||ribosomal protein L10A [Heliconius melpomene cythera]	6E-143	
	rep_c11981	1191	96.95	>gi|342356339|gb|AEL28828.1||ribosomal protein L10A [Heliconius melpomene cythera]	1E-92	
BmRpL11	joint2_rep_c38	757	98.96	>gi|389608383|dbj|BAM17801.1||ribosomal protein L11 [Papilio xuthus]	1E-121	Full
	rep_c49534	717	96.40	>gi|389608383|dbj|BAM17801.1||ribosomal protein L11 [Papilio xuthus]	5E-91	Partial
BmRpL12	rep_c50989	646	95.12	>gi|16566713|gb|AAL26576.1|AF429974_1|ribosomal protein L12 [Spodoptera frugiperda]	1E-109	Full
	joint2_rep_c11218	691	95.12	>gi|16566713|gb|AAL26576.1|AF429974_1|ribosomal protein L12 [Spodoptera frugiperda]	2E-109	
	rep_c50524	700	95.12	>gi|16566713|gb|AAL26576.1|AF429974_1|ribosomal protein L12 [Spodoptera frugiperda]	2E-109	
	joint2_rep_c181	703	95.12	>gi|16566713|gb|AAL26576.1|AF429974_1|ribosomal protein L12 [Spodoptera frugiperda]	2E-109	
	rep_c48909	690	93.87	>gi|16566713|gb|AAL26576.1|AF429974_1|ribosomal protein L12 [Spodoptera frugiperda]	1E-106	
BmRpL13	joint2_rep_c387	935	94.57	>gi|56463257|gb|AAV91770.1||ribosomal protein L13 [Helicoverpa zea]	7E-142	Full
	rep_c11806	852	93.51	>gi|56463257|gb|AAV91770.1||ribosomal protein L13 [Helicoverpa zea]	6E-105	
	rep_c21090	684	87.40	>gi|21759389|sp|Q962U1.1|RL13_SPOFR|ribosomal protein L13 [Spodoptera frugiperda]	1E-76	Partial
BmRpL13A	joint2_rep_c10983	787	91.18	>gi|31340318|sp|Q962U0.1|RL13A_SPOFR|ribosomal protein L13A [Spodoptera frugiperda]	7E-135	Full
	joint2_rep_c605	883	91.18	>gi|31340318|sp|Q962U0.1|RL13A_SPOFR|ribosomal protein L13A [Spodoptera frugiperda]	2E-134	
BmRpL14	rep_c51115	686	91.03	>gi|15213764|gb|AAK92157.1|AF400185_1|ribosomal protein L14 [Spodoptera frugiperda]	1E-101	Full
	joint2_rep_c50	773	91.03	>gi|15213764|gb|AAK92157.1|AF400185_1|ribosomal protein L14 [Spodoptera frugiperda]	4E-101	
	c11121	1938	91.72	>gi|15213764|gb|AAK92157.1|AF400185_1|ribosomal protein L14 [Spodoptera frugiperda]	9E-97	
BmRpL15	joint2_rep_c637	874	96.08	>gi|27462516|gb|AAO15464.1|AF441167_1|60S ribosomal protein L15 [Spodoptera frugiperda]	6E-114	Full
BmRpL17	joint2_rep_c160	786	97.40	>gi|268306390|gb|ACY95316.1||ribosomal protein L17 [Manduca sexta]	2E-105	Full
	rep_c50465	660	97.39	>gi|268306390|gb|ACY95316.1||ribosomal protein L17 [Manduca sexta]	2E-105	
	rep_c48691	715	96.24	>gi|268306390|gb|ACY95316.1||ribosomal protein L17 [Manduca sexta]	6E-88	Partial
	rep_c42055	675	94.74	>gi|268306390|gb|ACY95316.1||ribosomal protein L17 [Manduca sexta]	2E-85	

BmRpL18	rep_c27514	631	91.26	>gi|74938646|sp|Q962Y8.1|RL18_SPOFR|ribosomal protein L18 [Spodoptera frugiperda]	1E-110	Full
	joint2_rep_c427	701	91.26	>gi|74938646|sp|Q962Y8.1|RL18_SPOFR|ribosomal protein L18 [Spodoptera frugiperda]	2E-110	
BmRpL18A	joint2_rep_c1977	682	94.92	>gi|21362850|sp|Q8WQI7.1|RL18A_SPOFR|ribosomal protein L18A [Spodoptera frugiperda]	2E-125	Full
	rep_c124	781	94.92	>gi|21362850|sp|Q8WQI7.1|RL18A_SPOFR|ribosomal protein L18A [Spodoptera frugiperda]	7E-125	
BmRpL19	joint2_rep_c435	779	96.50	>gi|342356421|gb|AEL28869.1||ribosomal protein L19 [Heliconius melpomene cythera]	3E-96	Full
BmRpL21	joint2_rep_c509	681	94.34	>gi|15213768|gb|AAK92159.1|AF400187_1|ribosomal protein L21 [Spodoptera frugiperda]	1E-109	Full
	rep_c50994	696	93.33	>gi|15213768|gb|AAK92159.1|AF400187_1|ribosomal protein L21 [Spodoptera frugiperda]	6E-90	
BmRpL22	rep_c49023	562	92.52	>gi|15213770|gb|AAK92160.1|AF400188_1|ribosomal protein L22 [Spodoptera frugiperda]	6E-41	Full
	joint2_rep_c183	654	93.20	>gi|15213770|gb|AAK92160.1|AF400188_1|ribosomal protein L22 [Spodoptera frugiperda]	5E-45	
	rep_c30401	641	92.50	>gi|15213770|gb|AAK92160.1|AF400188_1|ribosomal protein L22 [Spodoptera frugiperda]	4E-32	Partial
	c26675	799	90.32	>gi|15213770|gb|AAK92160.1|AF400188_1|ribosomal protein L22 [Spodoptera frugiperda]	1E-28	
BmRpL23	rep_c51254	490	100.00	>gi|112984274|ref|NP_001037227.1||ribosomal protein L23 [Bombyx mori]	5E-93	Full
	rep_c48973	496	100.00	>gi|112984274|ref|NP_001037227.1||ribosomal protein L23 [Bombyx mori]	3E-87	
	rep_c51125	500	100.00	>gi|112984274|ref|NP_001037227.1||ribosomal protein L23 [Bombyx mori]	5E-93	
	rep_c51122	506	100.00	>gi|112984274|ref|NP_001037227.1||ribosomal protein L23 [Bombyx mori]	6E-93	
	rep_c51097	512	100.00	>gi|112984274|ref|NP_001037227.1||ribosomal protein L23 [Bombyx mori]	6E-93	
	rep_c50138	511	100.00	>gi|112984274|ref|NP_001037227.1||ribosomal protein L23 [Bombyx mori]	6E-93	
	rep_c47999	534	100.00	>gi|112984274|ref|NP_001037227.1||ribosomal protein L23 [Bombyx mori]	8E-93	
	rep_c51104	506	100.00	>gi|112984274|ref|NP_001037227.1||ribosomal protein L23 [Bombyx mori]	6E-93	
	rep_c48060	573	100.00	>gi|112984274|ref|NP_001037227.1||ribosomal protein L23 [Bombyx mori]	1E-92	
	rep_c48622	508	99.29	>gi|112984274|ref|NP_001037227.1||ribosomal protein L23 [Bombyx mori]	2E-92	
	rep_c48976	503	99.29	>gi|112984274|ref|NP_001037227.1||ribosomal protein L23 [Bombyx mori]	4E-83	
	rep_c51106	483	99.29	>gi|112984274|ref|NP_001037227.1||ribosomal protein L23 [Bombyx mori]	3E-92	
	rep_c51198	492	99.29	>gi|112984274|ref|NP_001037227.1||ribosomal protein L23 [Bombyx mori]	3E-92	
	rep_c51209	491	99.29	>gi|112984274|ref|NP_001037227.1||ribosomal protein L23 [Bombyx mori]	4E-92	
	joint2_rep_c11316	511	99.29	>gi|112984274|ref|NP_001037227.1||ribosomal protein L23 [Bombyx mori]	2E-81	
	rep_c48562	529	99.29	>gi|112984274|ref|NP_001037227.1||ribosomal protein L23 [Bombyx mori]	6E-92	
	rep_c51210	538	98.57	>gi|112984274|ref|NP_001037227.1||ribosomal protein L23 [Bombyx mori]	1E-91	
	rep_c48990	625	99.29	>gi|112984274|ref|NP_001037227.1||ribosomal protein L23 [Bombyx mori]	2E-91	
	rep_c48155	546	98.57	>gi|112984274|ref|NP_001037227.1||ribosomal protein L23 [Bombyx mori]	3E-91	
	rep_c49762	529	98.57	>gi|112984274|ref|NP_001037227.1||ribosomal protein L23 [Bombyx mori]	5E-91	
	rep_c49365	502	98.57	>gi|112984274|ref|NP_001037227.1||ribosomal protein L23 [Bombyx mori]	2E-90	
	rep_c50687	511	97.86	>gi|112984274|ref|NP_001037227.1||ribosomal protein L23 [Bombyx mori]	3E-90	
	rep_c50574	532	97.86	>gi|112984274|ref|NP_001037227.1||ribosomal protein L23 [Bombyx mori]	3E-89	
	joint2_rep_c11328	642	100.00	>gi|112984274|ref|NP_001037227.1||ribosomal protein L23 [Bombyx mori]	2E-89	
	rep_c35365	542	95.00	>gi|112984274|ref|NP_001037227.1||ribosomal protein L23 [Bombyx mori]	1E-86	
	rep_c49750	543	92.86	>gi|112984274|ref|NP_001037227.1||ribosomal protein L23 [Bombyx mori]	3E-78	
	rep_c51113	513	98.18	>gi|112984274|ref|NP_001037227.1||ribosomal protein L23 [Bombyx mori]	2E-68	Partial
BmRpL24	rep_c15435	472	98.00	>gi|52783262|sp|Q962T5.1|RL24_SPOFR|ribosomal protein L24 [Spodoptera frugiperda]	3E-61	Partial
	joint2_rep_c498	676	97.00	>gi|52783262|sp|Q962T5.1|RL24_SPOFR|ribosomal protein L24 [Spodoptera frugiperda]	5E-53	
	rep_c21299	629	97.96	>gi|52783262|sp|Q962T5.1|RL24_SPOFR|ribosomal protein L24 [Spodoptera frugiperda]	1E-55	
BmRpL26	joint2_rep_c2725	1275	95.27	>gi|15213774|gb|AAK92162.1|AF400190_1|ribosomal protein L26 [Spodoptera frugiperda]	2E-71	Full
	rep_c51502	439	94.26	>gi|15213774|gb|AAK92162.1|AF400190_1|ribosomal protein L26 [Spodoptera frugiperda]	2E-63	
BmRpL27	joint2_rep_c11179	872	97.76	>gi|15213776|gb|AAK92163.1|AF400191_1|ribosomal protein L27 [Spodoptera frugiperda]	8E-84	Full
	joint2_rep_c918	628	97.01	>gi|15213776|gb|AAK92163.1|AF400191_1|ribosomal protein L27 [Spodoptera frugiperda]	1E-83	
	rep_c17994	738	75.68	>gi|15213776|gb|AAK92163.1|AF400191_1|ribosomal protein L27 [Spodoptera frugiperda]	1E-61	
BmRpL27A	rep_c50902	466	86.96	>gi|15213766|gb|AAK92158.1|AF400186_1|ribosomal protein L27A [Spodoptera frugiperda]	1E-66	Partial
	joint2_rep_c513	572	91.26	>gi|15213766|gb|AAK92158.1|AF400186_1|ribosomal protein L27A [Spodoptera frugiperda]	7E-56	
BmRpL28	rep_c49599	564	92.86	>gi|24418650|sp|Q962T2.1|RL28_SPOFR|ribosomal protein L28 [Spodoptera frugiperda]	2E-70	Full
	joint2_rep_c279	601	92.86	>gi|24418650|sp|Q962T2.1|RL28_SPOFR|ribosomal protein L28 [Spodoptera frugiperda]	3E-70	
BmRpL29	rep_c48506	382	96.49	>gi|16566716|gb|AAL26577.1|AF429975_1|ribosomal protein L29 [Spodoptera frugiperda]	5E-34	Partial
	rep_c5271	598	96.49	>gi|16566716|gb|AAL26577.1|AF429975_1|ribosomal protein L29 [Spodoptera frugiperda]	5E-33	
BmRpL30	joint2_rep_c290	607	99.10	>gi|17368248|sp|P58375.1|RL30_SPOFR|ribosomal protein L30 [Spodoptera frugiperda]	2E-72	Full
	rep_c5538	585	93.06	>gi|17368248|sp|P58375.1|RL30_SPOFR|ribosomal protein L30 [Spodoptera frugiperda]	8E-53	Partial
	rep_c49545	506	93.06	>gi|17368248|sp|P58375.1|RL30_SPOFR|ribosomal protein L30 [Spodoptera frugiperda]	6E-53	
	rep_c52829	610	72.32	>gi|17368248|sp|P58375.1|RL30_SPOFR|ribosomal protein L30 [Spodoptera frugiperda]	5E-45	Full
	rep_c24508	504	54.55	>gi|555954209|ref|XP_005889273.1||PREDICTED: 60S ribosomal protein L30-like [Bos mutus]	2E-20	Partial
BmRpL31	joint2_rep_c456	529	99.19	>gi|51701794|sp|Q7KF90.1|RL31_SPOFR|ribosomal protein L31 [Spodoptera frugiperda]	7E-73	Full
	rep_c2075	760	99.19	>gi|51701794|sp|Q7KF90.1|RL31_SPOFR|ribosomal protein L31 [Spodoptera frugiperda]	1E-71	
BmRpL32	joint2_rep_c610	579	97.76	>gi|51701837|sp|Q962T1.1|RL32_SPOFR|ribosomal protein L32 [Spodoptera frugiperda]	3E-90	Full
BmRpL34	rep_c49906	558	99.04	>gi|18253053|gb|AAL62471.1||ribosomal protein L34 [Spodoptera frugiperda]	2E-73	Full

	joint2_rep_c194	633	99.04	>gi|18253053|gb|AAL62471.1||ribosomal protein L34 [Spodoptera frugiperda]	8E-77	Full
BmRpL34	rep_c50786	478	92.55	>gi|18253053|gb|AAL62471.1||ribosomal protein L34 [Spodoptera frugiperda]	3E-64	Partial
	rep_c36933	360	97.56	>gi|18253053|gb|AAL62471.1||ribosomal protein L34 [Spodoptera frugiperda]	5E-50	
	joint2_rep_c2968	465	99.19	>gi|357612425|gb|EHJ67994.1||ribosomal protein L35 [Danaus plexippus]	3E-65	
BmRpL35	joint2_rep_c93	580	99.19	>gi|357612425|gb|EHJ67994.1||ribosomal protein L35 [Danaus plexippus]	1E-64	Full
	rep_c50493	690	99.19	>gi|357612425|gb|EHJ67994.1||ribosomal protein L35 [Danaus plexippus]	2E-64	
	joint2_rep_c4133	819	99.19	>gi|112984164|ref|NP_001037241.1||ribosomal protein L35 [Bombyx mori]	4E-63	
BmRpL35A	joint2_rep_c2989	657	89.26	>gi|15213788|gb|AAK92169.1|AF400197_1|ribosomal protein L35A [Spodoptera frugiperda]	7E-80	Partial
	joint2_rep_c257	1020	89.26	>gi|15213788|gb|AAK92169.1|AF400197_1|ribosomal protein L35A [Spodoptera frugiperda]	1E-78	
	joint2_rep_c11283	522	95.41	>gi|15213790|gb|AAK92170.1|AF400198_1|ribosomal protein L36A [Spodoptera frugiperda]	3E-67	
BmRpL36	rep_c50374	587	95.41	>gi|15213790|gb|AAK92170.1|AF400198_1|ribosomal protein L36A [Spodoptera frugiperda]	7E-67	Full
	joint2_rep_c2994	499	94.50	>gi|15213790|gb|AAK92170.1|AF400198_1|ribosomal protein L36A [Spodoptera frugiperda]	2E-66	
	joint2_rep_c11293	422	99.04	>gi|15213804|gb|AAK92177.1|AF400205_1|ribosomal protein L44 [Spodoptera frugiperda]	2E-50	
	rep_c49419	426	99.04	>gi|15213804|gb|AAK92177.1|AF400205_1|ribosomal protein L44 [Spodoptera frugiperda]	2E-50	
	joint2_rep_c10969	473	99.04	>gi|15213804|gb|AAK92177.1|AF400205_1|ribosomal protein L44 [Spodoptera frugiperda]	3E-50	
	rep_c49011	521	97.09	>gi|15213804|gb|AAK92177.1|AF400205_1|ribosomal protein L44 [Spodoptera frugiperda]	4E-59	Full
BmRpL36A	rep_c50516	415	96.15	>gi|15213804|gb|AAK92177.1|AF400205_1|ribosomal protein L44 [Spodoptera frugiperda]	9E-39	
	joint2_rep_c11329	416	94.87	>gi|15213804|gb|AAK92177.1|AF400205_1|ribosomal protein L44 [Spodoptera frugiperda]	4E-38	
	joint2_rep_c11331	435	94.87	>gi|15213804|gb|AAK92177.1|AF400205_1|ribosomal protein L44 [Spodoptera frugiperda]	5E-38	
	rep_c51193	450	92.31	>gi|15213804|gb|AAK92177.1|AF400205_1|ribosomal protein L44 [Spodoptera frugiperda]	9E-37	
	rep_c49515	438	98.21	>gi|291225924|ref|XP_002732947.1||PREDICTED: ribosomal protein L36a-like protein-like [Saccoglossus kowalevskii]	1E-31	Partial
	rep_c52739	347	98.21	>gi|15213804|gb|AAK92177.1|AF400205_1|ribosomal protein L44 [Spodoptera frugiperda]	7E-33	
	rep_c50213	412	98.92	>gi|20139580|sp|Q962S7.3|RL37_SPOFR|ribosomal protein L37 [Spodoptera frugiperda]	2E-52	
	joint2_rep_c306	419	98.92	>gi|20139580|sp|Q962S7.3|RL37_SPOFR|ribosomal protein L37 [Spodoptera frugiperda]	3E-52	
BmRpL37	rep_c48906	421	97.85	>gi|20139580|sp|Q962S7.3|RL37_SPOFR|ribosomal protein L37 [Spodoptera frugiperda]	1E-50	Full
	rep_c32481	858	98.92	>gi|20139580|sp|Q962S7.3|RL37_SPOFR|ribosomal protein L37 [Spodoptera frugiperda]	3E-50	
	rep_c48546	482	96.77	>gi|20139580|sp|Q962S7.3|RL37_SPOFR|ribosomal protein L37 [Spodoptera frugiperda]	4E-39	
	joint2_rep_c3555	459	100.00	>gi|112984128|ref|NP_001037249.1||ribosomal protein L37A [Bombyx mori]	1E-58	
	rep_c51151	388	100.00	>gi|112984128|ref|NP_001037249.1||ribosomal protein L37A [Bombyx mori]	5E-59	
BmRpL37A	rep_c51235	414	100.00	>gi|112984128|ref|NP_001037249.1||ribosomal protein L37A [Bombyx mori]	7E-59	Full
	joint2_rep_c11073	457	100.00	>gi|112984128|ref|NP_001037249.1||ribosomal protein L37A [Bombyx mori]	1E-58	
	rep_c49244	377	98.89	>gi|112984128|ref|NP_001037249.1||ribosomal protein L37A [Bombyx mori]	2E-56	
	rep_c51074	595	96.63	>gi|112984128|ref|NP_001037249.1||ribosomal protein L37A [Bombyx mori]	1E-54	
	rep_c50135	324	98.57	>gi|357611939|gb|EHJ67727.1||60S ribosomal protein L38 [Danaus plexippus]	4E-41	
	rep_c49945	325	98.57	>gi|357611939|gb|EHJ67727.1||60S ribosomal protein L38 [Danaus plexippus]	4E-41	
	rep_c49891	327	98.57	>gi|357611939|gb|EHJ67727.1||60S ribosomal protein L38 [Danaus plexippus]	5E-41	
	rep_c48868	329	98.57	>gi|357611939|gb|EHJ67727.1||60S ribosomal protein L38 [Danaus plexippus]	5E-41	
	rep_c49486	334	98.57	>gi|357611939|gb|EHJ67727.1||60S ribosomal protein L38 [Danaus plexippus]	5E-41	
	rep_c49625	347	98.57	>gi|357611939|gb|EHJ67727.1||60S ribosomal protein L38 [Danaus plexippus]	6E-41	
	rep_c50156	389	98.57	>gi|357611939|gb|EHJ67727.1||60S ribosomal protein L38 [Danaus plexippus]	1E-40	
	joint2_rep_c11313	392	98.57	>gi|357611939|gb|EHJ67727.1||60S ribosomal protein L38 [Danaus plexippus]	1E-40	
	joint2_rep_c11185	423	98.57	>gi|357611939|gb|EHJ67727.1||60S ribosomal protein L38 [Danaus plexippus]	1E-40	
	joint2_rep_c11297	464	98.57	>gi|357611939|gb|EHJ67727.1||60S ribosomal protein L38 [Danaus plexippus]	2E-40	
	rep_c49884	467	98.57	>gi|74910327|sp|Q6F450.1|RL38_PLUXY|Ribosomal protein L38 [Plutella xylostella]	8E-40	
BmRpL38	rep_c29119	472	98.57	>gi|74910327|sp|Q6F450.1|RL38_PLUXY|Ribosomal protein L38 [Plutella xylostella]	8E-40	Full
	rep_c38287	475	98.57	>gi|357611939|gb|EHJ67727.1||60S ribosomal protein L38 [Danaus plexippus]	3E-40	
	joint2_rep_c11076	480	98.57	>gi|357611939|gb|EHJ67727.1||60S ribosomal protein L38 [Danaus plexippus]	3E-40	
	joint2_rep_c10966	488	98.57	>gi|357611939|gb|EHJ67727.1||60S ribosomal protein L38 [Danaus plexippus]	3E-40	
	joint2_rep_c11306	519	98.57	>gi|357611939|gb|EHJ67727.1||60S ribosomal protein L38 [Danaus plexippus]	5E-40	
	rep_c38931	542	98.57	>gi|357611939|gb|EHJ67727.1||60S ribosomal protein L38 [Danaus plexippus]	6E-40	
	rep_c49765	334	97.14	>gi|357611939|gb|EHJ67727.1||60S ribosomal protein L38 [Danaus plexippus]	3E-40	
	rep_c49568	326	97.14	>gi|357611939|gb|EHJ67727.1||60S ribosomal protein L38 [Danaus plexippus]	5E-40	
	rep_c50154	325	97.14	>gi|357611939|gb|EHJ67727.1||60S ribosomal protein L38 [Danaus plexippus]	7E-40	
	rep_c48950	496	98.55	>gi|74910327|sp|Q6F450.1|RL38_PLUXY|Ribosomal protein L38 [Plutella xylostella]	6E-39	
	rep_c49678	384	95.71	>gi|357611939|gb|EHJ67727.1||60S ribosomal protein L38 [Danaus plexippus]	4E-39	
	rep_c49095	346	94.29	>gi|357611939|gb|EHJ67727.1||60S ribosomal protein L38 [Danaus plexippus]	1E-37	
	rep_c49664	275	100.00	>gi|350423620|ref|XP_003493538.1||PREDICTED: 60S ribosomal protein L39-like [Bombus impatiens]	4E-30	
	rep_c51073	272	100.00	>gi|350423620|ref|XP_003493538.1||PREDICTED: 60S ribosomal protein L39-like [Bombus impatiens]	4E-30	
BmRpL39	rep_c50036	275	100.00	>gi|350423620|ref|XP_003493538.1||PREDICTED: 60S ribosomal protein L39-like [Bombus impatiens]	4E-30	Full
	rep_c49431	275	100.00	>gi|350423620|ref|XP_003493538.1||PREDICTED: 60S ribosomal protein L39-like [Bombus impatiens]	4E-30	
	rep_c49685	278	100.00	>gi|350423620|ref|XP_003493538.1||PREDICTED: 60S ribosomal protein L39-like [Bombus impatiens]	4E-30	

BmRpL39	rep_c49872	277	100.00	>gi|350423620|ref|XP_003493538.1||PREDICTED: 60S ribosomal protein L39-like [Bombus impatiens]	4E-30	Full
	rep_c49536	276	100.00	>gi|350423620|ref|XP_003493538.1||PREDICTED: 60S ribosomal protein L39-like [Bombus impatiens]	4E-30	
	rep_c48660	276	100.00	>gi|350423620|ref|XP_003493538.1||PREDICTED: 60S ribosomal protein L39-like [Bombus impatiens]	4E-30	
	rep_c49204	276	100.00	>gi|350423620|ref|XP_003493538.1||PREDICTED: 60S ribosomal protein L39-like [Bombus impatiens]	4E-30	
	rep_c49790	297	100.00	>gi|350423620|ref|XP_003493538.1||PREDICTED: 60S ribosomal protein L39-like [Bombus impatiens]	5E-30	
	rep_c51078	300	100.00	>gi|350423620|ref|XP_003493538.1||PREDICTED: 60S ribosomal protein L39-like [Bombus impatiens]	5E-30	
	rep_c49635	303	100.00	>gi|350423620|ref|XP_003493538.1||PREDICTED: 60S ribosomal protein L39-like [Bombus impatiens]	5E-30	
	rep_c49938	306	100.00	>gi|350423620|ref|XP_003493538.1||PREDICTED: 60S ribosomal protein L39-like [Bombus impatiens]	6E-30	
	rep_c48934	368	100.00	>gi|350423620|ref|XP_003493538.1||PREDICTED: 60S ribosomal protein L39-like [Bombus impatiens]	1E-29	
	rep_c49196	423	100.00	>gi|350423620|ref|XP_003493538.1||PREDICTED: 60S ribosomal protein L39-like [Bombus impatiens]	2E-29	
	rep_c49690	276	98.04	>gi|350423620|ref|XP_003493538.1||PREDICTED: 60S ribosomal protein L39-like [Bombus impatiens]	3E-29	
	rep_c49373	274	98.04	>gi|350423620|ref|XP_003493538.1||PREDICTED: 60S ribosomal protein L39-like [Bombus impatiens]	2E-22	
	rep_c49470	276	98.04	>gi|350423620|ref|XP_003493538.1||PREDICTED: 60S ribosomal protein L39-like [Bombus impatiens]	2E-29	
	rep_c49425	278	98.04	>gi|350423620|ref|XP_003493538.1||PREDICTED: 60S ribosomal protein L39-like [Bombus impatiens]	3E-29	
	rep_c49943	361	98.04	>gi|350423620|ref|XP_003493538.1||PREDICTED: 60S ribosomal protein L39-like [Bombus impatiens]	4E-29	
	joint2_rep_c11299	300	98.04	>gi|350423620|ref|XP_003493538.1||PREDICTED: 60S ribosomal protein L39-like [Bombus impatiens]	4E-29	
	rep_c50084	304	98.04	>gi|350423620|ref|XP_003493538.1||PREDICTED: 60S ribosomal protein L39-like [Bombus impatiens]	4E-29	
	rep_c50243	306	98.04	>gi|350423620|ref|XP_003493538.1||PREDICTED: 60S ribosomal protein L39-like [Bombus impatiens]	4E-29	
	rep_c49758	275	96.08	>gi|350423620|ref|XP_003493538.1||PREDICTED: 60S ribosomal protein L39-like [Bombus impatiens]	2E-28	
	rep_c49582	280	96.08	>gi|350423620|ref|XP_003493538.1||PREDICTED: 60S ribosomal protein L39-like [Bombus impatiens]	3E-28	
	joint2_rep_c11260	392	96.08	>gi|350423620|ref|XP_003493538.1||PREDICTED: 60S ribosomal protein L39-like [Bombus impatiens]	1E-27	
	rep_c49575	276	96.08	>gi|350423620|ref|XP_003493538.1||PREDICTED: 60S ribosomal protein L39-like [Bombus impatiens]	2E-28	
	rep_c50348	279	94.12	>gi|350423620|ref|XP_003493538.1||PREDICTED: 60S ribosomal protein L39-like [Bombus impatiens]	5E-26	
	rep_c51258	271	96.97	>gi|350423620|ref|XP_003493538.1||PREDICTED: 60S ribosomal protein L39-like [Bombus impatiens]	1E-18	Partial
	rep_c21794	761	100.00	>gi|307206146|gb|EFN84226.1||60S ribosomal protein L39 [Harpegnathos saltator]	5E-15	
	rep_c51124	271	93.10	>gi|546681941|gb|ERL91937.1||hypothetical protein D910_09260 [Dendroctonus ponderosae]	4E-15	
	rep_c50578	287	92.86	>gi|546681941|gb|ERL91937.1||hypothetical protein D910_09260 [Dendroctonus ponderosae]	9E-14	
BmRpL40	joint2_rep_c546	694	98.39	>gi|15213800|gb|AAK92175.1|AF400203_1|ribosomal protein L40 [Spodoptera frugiperda]	2E-78	Full
	joint2_rep_c98	872	95.95	>gi|15213800|gb|AAK92175.1|AF400203_1|ribosomal protein L40 [Spodoptera frugiperda]	2E-59	Partial
	rep_c51284	377	98.98	>gi|15213800|gb|AAK92175.1|AF400203_1|ribosomal protein L40 [Spodoptera frugiperda]	5E-62	
	rep_c10476	466	84.62	>gi|300422605|dbj|BAJ10868.1||ubiquitin C [Callithrix jacchus]	2E-49	
	rep_c22330	1029	97.40	>gi|302393750|sp|P29504.2|RS27A_MANSE|ribosomal protein S27A [Manduca sexta]	7E-80	
	rep_c19288	1153	70.09	>gi|148237866|ref|NP_001079589.1||Polyubiquitin-C-like [Xenopus laevis]	0.0	Full
	rep_c19288	1153	98.68	>gi|148237866|ref|NP_001079589.1||Polyubiquitin-C-like [Xenopus laevis]	0.0	Partial
	rep_c19288	1153	98.68	>gi|148237866|ref|NP_001079589.1||Polyubiquitin-C-like [Xenopus laevis]	0.0	
	rep_c19288	1153	98.68	>gi|148237866|ref|NP_001079589.1||Polyubiquitin-C-like [Xenopus laevis]	0.0	
	joint2_rep_c6277	1347	98.68	>gi|148687613|gb|EDL19560.1||ubiquitin C, isoform CRA_a [Mus musculus]	0.0	
	joint2_rep_c6277	1347	98.68	>gi|148687613|gb|EDL19560.1||ubiquitin C, isoform CRA_a [Mus musculus]	0.0	
	joint2_rep_c6277	1347	88.51	>gi|148687613|gb|EDL19560.1||ubiquitin C, isoform CRA_a [Mus musculus]	0.0	
	joint2_rep_c6277	1347	97.37	>gi|148687613|gb|EDL19560.1||ubiquitin C, isoform CRA_a [Mus musculus]	0.0	
	joint2_rep_c6277	1347	85.33	>gi|148687613|gb|EDL19560.1||ubiquitin C, isoform CRA_a [Mus musculus]	0.0	
	joint2_rep_c1513	1726	98.68	>gi|444434921|dbj|BAM77035.1||ubiquitin C [Homo sapiens]	4E-161	
	joint2_rep_c1513	1726	98.68	>gi|444434921|dbj|BAM77035.1||ubiquitin C [Homo sapiens]	4E-161	
	rep_c1554	1741	98.68	>gi|557760363|ref|XP_005179902.1||PREDICTED: polyubiquitin-C-like isoform X1 [Musca domestica]	0.0	
	rep_c1554	1741	98.68	>gi|557760363|ref|XP_005179902.1||PREDICTED: polyubiquitin-C-like isoform X1 [Musca domestica]	0.0	
	rep_c1554	1741	89.66	>gi|557760363|ref|XP_005179902.1||PREDICTED: polyubiquitin-C-like isoform X1 [Musca domestica]	0.0	
	joint2_rep_c145	698	93.51	>gi|302393750|sp|P29504.2|RS27A_MANSE|ribosomal protein S27A [Manduca sexta]	9E-78	
	rep_c8371	977	92.59	>gi|302393750|sp|P29504.2|RS27A_MANSE|ribosomal protein S27A [Manduca sexta]	2E-75	
	joint2_rep_c6757	658	98.53	>gi|511976401|ref|XP_004806134.1||PREDICTED: polyubiquitin-B-like [Mustela putorius furo]	5E-73	
	rep_c6835	524	98.48	>gi|402904829|ref|XP_003915241.1||PREDICTED: ubiquitin-60S ribosomal protein L40 isoform 5 [Papio anubis]	4E-37	
	rep_c1158	557	57.89	>gi|512896698|ref|XP_004923973.1||PREDICTED: NEDD8-like [Bombyx mori]	6E-27	
	rep_c9117	784	57.89	>gi|512896698|ref|XP_004923973.1||PREDICTED: NEDD8-like [Bombyx mori]	4E-26	
	joint2_rep_c1094	786	57.89	>gi|512896698|ref|XP_004923973.1||PREDICTED: NEDD8-like [Bombyx mori]	4E-26	
	rep_c10105	802	57.89	>gi|512896698|ref|XP_004923973.1||PREDICTED: NEDD8-like [Bombyx mori]	4E-26	
	c47482	1158	56.58	>gi|512896698|ref|XP_004923973.1||PREDICTED: NEDD8-like [Bombyx mori]	2E-24	
BmRpSA	joint2_rep_c123	1136	88.97	>gi|389610689|dbj|BAM18956.1||stubarista [Papilio polytes]	5E-155	Full
BmRpS2	rep_c34010	1198	99.07	>gi|27260896|gb|AAN86048.1||ribosomal protein S2 [Spodoptera frugiperda]	3E-136	Full
	joint2_rep_c519	1597	99.08	>gi|27260896|gb|AAN86048.1||ribosomal protein S2 [Spodoptera frugiperda]	5E-135	
BmRpS3	joint2_rep_c31	1787	99.13	>gi|315115341|gb|ADT80643.1||ribosomal protein S3 [Euphydryas aurinia]	6E-154	Full
BmRpS3A	joint2_rep_c39	1086	97.13	>gi|74938351|sp|Q95V35.1|RS3A_SPOFR|ribosomal protein S3A [Spodoptera frugiperda]	1E-157	Full
	c47593	968	91.59	>gi|74938351|sp|Q95V35.1|RS3A_SPOFR|ribosomal protein S3A [Spodoptera frugiperda]	8E-138	

BmRpS3A	joint2_rep_c2087	944	97.47	>gi|74938351|sp|Q95V35.1|RS3A_SPOFR|ribosomal protein S3A [Spodoptera frugiperda]	2E-137	Partial
BmRpS4	rep_c107	979	97.34	>gi|74844658|sp|Q95V34.1|RS4_SPOFR|ribosomal protein S4 [Spodoptera frugiperda]	0.0	Full
	rep_c6620	1155	96.58	>gi|74844658|sp|Q95V34.1|RS4_SPOFR|ribosomal protein S4 [Spodoptera frugiperda]	0.0	
	joint2_rep_c69	1050	96.20	>gi|74844658|sp|Q95V34.1|RS4_SPOFR|ribosomal protein S4 [Spodoptera frugiperda]	0.0	
BmRpS5	joint2_rep_c1381	770	95.43	>gi|16566728|gb|AAL26581.1|AF429979_1|ribosomal protein S5 [Spodoptera frugiperda]	3E-151	Full
	joint2_rep_c6898	849	94.52	>gi|16566728|gb|AAL26581.1|AF429979_1|ribosomal protein S5 [Spodoptera frugiperda]	7E-149	
BmRpS6	joint2_rep_c11151	924	95.26	>gi|20139899|sp|Q95V32.1|RS6_SPOFR|ribosomal protein S6 [Spodoptera frugiperda]	5E-157	Full
	joint2_rep_c11273	878	93.28	>gi|20139899|sp|Q95V32.1|RS6_SPOFR|ribosomal protein S6 [Spodoptera frugiperda]	3E-154	
	joint2_rep_c10943	888	95.26	>gi|20139899|sp|Q95V32.1|RS6_SPOFR|ribosomal protein S6 [Spodoptera frugiperda]	2E-140	
	joint2_rep_c11318	872	91.67	>gi|20139899|sp|Q95V32.1|RS6_SPOFR|ribosomal protein S6 [Spodoptera frugiperda]	7E-130	
	rep_c48341	880	96.77	>gi|20139899|sp|Q95V32.1|RS6_SPOFR|ribosomal protein S6 [Spodoptera frugiperda]	2E-126	Partial
	rep_c48887	533	94.34	>gi|20139899|sp|Q95V32.1|RS6_SPOFR|ribosomal protein S6 [Spodoptera frugiperda]	4E-84	
BmRpS7	rep_c22858	780	97.37	>gi|49036475|sp|Q962S0.1|RS7_SPOFR|ribosomal protein S7 [Spodoptera frugiperda]	2E-126	Full
	joint2_rep_c143	848	97.37	>gi|49036475|sp|Q962S0.1|RS7_SPOFR|ribosomal protein S7 [Spodoptera frugiperda]	4E-126	
	joint2_rep_c30	962	97.37	>gi|49036475|sp|Q962S0.1|RS7_SPOFR|ribosomal protein S7 [Spodoptera frugiperda]	2E-125	
BmRpS8	joint2_rep_c122	1295	95.67	>gi|54039568|sp|Q8WQI5.1|RS8_SPOFR|ribosomal protein S8 [Spodoptera frugiperda]	2E-142	Full
BmRpS9	joint2_rep_c738	751	99.48	>gi|315115339|gb|ADT80642.1||ribosomal protein S9 [Euphydryas aurinia]	3E-123	Full
	joint2_rep_c2445	794	26.61	>gi|357620617|gb|EHJ72757.1||ribosomal protein S9 [Heliconius melpomene cythera]	3E-116	Partial
BmRpS10	rep_c9489	650	92.45	>gi|20140136|sp|Q962R9.1|RS10_SPOFR|ribosomal protein S10 [Spodoptera frugiperda]	5E-102	Full
	joint2_rep_c2208	669	89.47	>gi|20140136|sp|Q962R9.1|RS10_SPOFR|ribosomal protein S10 [Spodoptera frugiperda]	1E-75	Partial
	rep_c22141	758	85.38	>gi|20140136|sp|Q962R9.1|RS10_SPOFR|ribosomal protein S10 [Spodoptera frugiperda]	8E-57	
BmRpS11-1	joint2_rep_c105	660	87.41	>gi|14318169|gb|AAK59928.1|AF379640_1|ribosomal protein S11 [Heliothis virescens]	3E-95	Full
	joint2_rep_c2539	575	97.78	>gi|164420683|ref|NP_001106708.1||ribosomal protein S11 isoform 1 [Bombyx mori]	2E-72	Partial
	rep_c22167	741	96.67	>gi|15213810|gb|AAK92180.1|AF400208_1|ribosomal protein S11-1 [Manduca sexta]	6E-56	
BmRpS11-2	joint2_rep_c105	660	96.40	>gi|14318169|gb|AAK59928.1|AF379640_1|ribosomal protein S11 [Heliothis virescens]	3E-95	Full
	joint2_rep_c2539	575	97.78	>gi|164420683|ref|NP_001106708.1||ribosomal protein S11 isoform 1 [Bombyx mori]	2E-72	Partial
	rep_c22167	741	96.67	>gi|15213810|gb|AAK92180.1|AF400208_1|ribosomal protein S11-1 [Manduca sexta]	6E-56	
BmRpS12	joint2_rep_c150	620	95.59	>gi|15213812|gb|AAK92181.1|AF400209_1|ribosomal protein S12 [Spodoptera frugiperda]	5E-90	Full
	rep_c32099	622	94.12	>gi|15213812|gb|AAK92181.1|AF400209_1|ribosomal protein S12 [Spodoptera frugiperda]	1E-88	
	rep_c27507	608	94.53	>gi|15213812|gb|AAK92181.1|AF400209_1|ribosomal protein S12 [Spodoptera frugiperda]	2E-84	
BmRpS13	rep_c25473	720	98.61	>gi|54039367|sp|Q962R6.3|RS13_SPOFR|ribosomal protein S13 [Spodoptera frugiperda]	9E-94	Full
	joint2_rep_c3392	682	97.30	>gi|54039367|sp|Q962R6.3|RS13_SPOFR|ribosomal protein S13 [Spodoptera frugiperda]	2E-75	Partial
	rep_c4873	647	97.30	>gi|54039367|sp|Q962R6.3|RS13_SPOFR|ribosomal protein S13 [Spodoptera frugiperda]	2E-75	
	rep_c30168	598	96.51	>gi|148237954|ref|NP_001080351.1||ribosomal protein S13 [Xenopus laevis]	9E-65	
BmRpS14	joint2_rep_c219	662	99.29	>gi|15213816|gb|AAK92183.1|AF400211_1|ribosomal protein S14 [Spodoptera frugiperda]	5E-71	Full
	rep_c50544	678	99.29	>gi|15213816|gb|AAK92183.1|AF400211_1|ribosomal protein S14 [Spodoptera frugiperda]	5E-71	
	joint2_rep_c1650	835	99.29	>gi|15213816|gb|AAK92183.1|AF400211_1|ribosomal protein S14 [Spodoptera frugiperda]	3E-70	
	rep_c20510	608	95.59	>gi|15213816|gb|AAK92183.1|AF400211_1|ribosomal protein S14 [Spodoptera frugiperda]	2E-63	
	joint2_rep_c29	1317	99.26	>gi|15213816|gb|AAK92183.1|AF400211_1|ribosomal protein S14 [Spodoptera frugiperda]	3E-64	
BmRpS15	joint2_rep_c460	696	98.64	>gi|15213818|gb|AAK92184.1|AF400212_1|ribosomal protein S15 [Spodoptera frugiperda]	2E-78	Full
	rep_c50916	556	92.52	>gi|15213818|gb|AAK92184.1|AF400212_1|ribosomal protein S15 [Spodoptera frugiperda]	2E-83	
BmRpS15A	joint2_rep_c3476	563	100.00	>gi|112982855|ref|NP_001037570.1||ribosomal protein S15A [Bombyx mori]	5E-87	Full
	joint2_rep_c725	591	100.00	>gi|112982855|ref|NP_001037570.1||ribosomal protein S15A [Bombyx mori]	7E-87	
	joint2_rep_c9364	664	100.00	>gi|112982855|ref|NP_001037570.1||ribosomal protein S15A [Bombyx mori]	2E-86	
BmRpS16	joint2_rep_c2803	613	98.01	>gi|54039446|sp|Q95V31.1|RS16_SPOFR|ribosomal protein S16 [Spodoptera frugiperda]	5E-101	Full
	joint2_rep_c239	753	98.01	>gi|54039446|sp|Q95V31.1|RS16_SPOFR|ribosomal protein S16 [Spodoptera frugiperda]	3E-100	
	joint2_rep_c311	738	92.41	>gi|54039446|sp|Q95V31.1|RS16_SPOFR|ribosomal protein S16 [Spodoptera frugiperda]	3E-95	
BmRpS17	joint2_rep_c51	603	100.00	>gi|112984008|ref|NP_001037267.1||ribosomal protein S17 [Bombyx mori]	8E-70	Full
	rep_c15799	721	100.00	>gi|112984008|ref|NP_001037267.1||ribosomal protein S17 [Bombyx mori]	3E-69	
	rep_c50845	518	91.67	>gi|112984008|ref|NP_001037267.1||ribosomal protein S17 [Bombyx mori]	5E-52	
BmRpS18	joint2_rep_c735	710	98.47	>gi|54039447|sp|Q962R1.1|RS18_SPOFR|ribosomal protein S18 [Spodoptera frugiperda]	2E-89	Full
	joint2_rep_c1580	1057	98.44	>gi|54039447|sp|Q962R1.1|RS18_SPOFR|ribosomal protein S18 [Spodoptera frugiperda]	1E-80	
	rep_c35330	745	92.31	>gi|54039447|sp|Q962R1.1|RS18_SPOFR|ribosomal protein S18 [Spodoptera frugiperda]	3E-60	
BmRpS19	joint2_rep_c94	620	95.52	>gi|15213826|gb|AAK92188.1|AF400216_1|ribosomal protein S19 [Spodoptera frugiperda]	2E-101	Full
	joint2_rep_c402	679	95.52	>gi|15213826|gb|AAK92188.1|AF400216_1|ribosomal protein S19 [Spodoptera frugiperda]	5E-101	
BmRpS20	joint2_rep_c11315	573	98.37	>gi|15213828|gb|AAK92189.1|AF400217_1|ribosomal protein S20 [Spodoptera frugiperda]	5E-79	Full
	joint2_rep_c10952	817	96.72	>gi|15213828|gb|AAK92189.1|AF400217_1|ribosomal protein S20 [Spodoptera frugiperda]	2E-75	
BmRpS21	rep_c3134	490	100.00	>gi|112984360|ref|NP_001037207.1||40S ribosomal protein S21 [Bombyx mori]	7E-52	Full
	rep_c7088	539	98.41	>gi|112984360|ref|NP_001037207.1||40S ribosomal protein S21 [Bombyx mori]	8E-36	Partial
	rep_c40652	632	100.00	>gi|112984360|ref|NP_001037207.1||40S ribosomal protein S21 [Bombyx mori]	6E-34	
BmRpS23	joint2_rep_c706	761	97.20	>gi|52000789|sp|Q6EV23.1|RS23_PAPDA|ribsomal protein S23e [Papilio dardanus]	4E-97	Full

BmRpS24	joint2_c1575	814	98.48	>gi|20140134|sp|Q962Q6.1|RS24_SPOFR|ribosomal protein S24 [Spodoptera frugiperda]	4E-79	Full
BmRpS25	rep_c45283	448	100.00	>gi|51316900|sp|Q962Q5.1|RS25_SPOFR|ribosomal protein S25 [Spodoptera frugiperda]	3E-54	Partial
	joint2_rep_c1339	505	100.00	>gi|51316900|sp|Q962Q5.1|RS25_SPOFR|ribosomal protein S25 [Spodoptera frugiperda]	2E-52	
	rep_c24571	532	97.40	>gi|51316900|sp|Q962Q5.1|RS25_SPOFR|ribosomal protein S25 [Spodoptera frugiperda]	2E-50	
BmRpS26	joint2_rep_c401	489	94.78	>gi|15213838|gb|AAK92194.1|AF400222_1|ribosomal protein S26 [Spodoptera frugiperda]	2E-77	Full
	joint2_rep_c11233	581	94.78	>gi|15213838|gb|AAK92194.1|AF400222_1|ribosomal protein S26 [Spodoptera frugiperda]	5E-77	
	rep_c50993	785	93.46	>gi|15213838|gb|AAK92194.1|AF400222_1|ribosomal protein S26 [Spodoptera frugiperda]	2E-68	
BmRpS27	rep_c28023	506	98.81	>gi|15213840|gb|AAK92195.1|AF400223_1|ribosomal protein S27 [Spodoptera frugiperda]	5E-56	Full
	rep_c44471	676	98.81	>gi|15213840|gb|AAK92195.1|AF400223_1|ribosomal protein S27 [Spodoptera frugiperda]	4E-55	
	rep_c14124	636	98.81	>gi|15213840|gb|AAK92195.1|AF400223_1|ribosomal protein S27 [Spodoptera frugiperda]	2E-55	
	rep_c282	647	98.81	>gi|15213840|gb|AAK92195.1|AF400223_1|ribosomal protein S27 [Spodoptera frugiperda]	3E-55	
BmRpS27A	rep_c22330	1029	98.71	>gi|302393750|sp|P29504.2|RS27A_MANSE|ribosomal protein S27A [Manduca sexta]	7E-80	Full
	rep_c8371	977	96.23	>gi|302393750|sp|P29504.2|RS27A_MANSE|ribosomal protein S27A [Manduca sexta]	2E-75	
	joint2_rep_c145	698	96.13	>gi|302393750|sp|P29504.2|RS27A_MANSE|ribosomal protein S27A [Manduca sexta]	9E-78	
BmRpS28	rep_c49362	415	100.00	>gi|112982910|ref|NP_001037680.1||40S ribosomal protein S28 [Bombyx mori]	4E-26	Partial
	joint2_rep_c271	502	100.00	>gi|112982910|ref|NP_001037680.1||40S ribosomal protein S28 [Bombyx mori]	8E-26	
	rep_c49889	510	100.00	>gi|112982910|ref|NP_001037680.1||40S ribosomal protein S28 [Bombyx mori]	9E-26	
	joint2_rep_c1795	406	98.04	>gi|112982910|ref|NP_001037680.1||40S ribosomal protein S28 [Bombyx mori]	4E-25	
BmRpS29	rep_c51888	389	98.21	>gi|54039495|sp|Q8WQI3.1|RS29_SPOFR|ribosomal protein S29 [Spodoptera frugiperda]	4E-36	Full
	rep_c38253	349	98.21	>gi|54039495|sp|Q8WQI3.1|RS29_SPOFR|ribosomal protein S29 [Spodoptera frugiperda]	2E-36	
	rep_c52698	387	98.21	>gi|54039495|sp|Q8WQI3.1|RS29_SPOFR|ribosomal protein S29 [Spodoptera frugiperda]	4E-36	
	joint2_rep_c1534	691	98.21	>gi|54039495|sp|Q8WQI3.1|RS29_SPOFR|ribosomal protein S29 [Spodoptera frugiperda]	9E-35	
BmRpS30	rep_c48559	590	72.73	>gi|15213844|gb|AAK92197.1|AF400225_1|ribosomal protein S30 [Spodoptera frugiperda]	2E-73	Full
	joint2_rep_c1566	959	71.76	>gi|15213844|gb|AAK92197.1|AF400225_1|ribosomal protein S30 [Spodoptera frugiperda]	1E-74	
	joint2_rep_c3178	603	60.87	>gi|15213844|gb|AAK92197.1|AF400225_1|ribosomal protein S30 [Spodoptera frugiperda]	9E-55	Partial
