# Supplementary material for: Establishment and analysis of a reference transcriptome for Spodoptera frugiperda
Source: BMC Genomics. 2014 Aug 23;15(1):704. doi: 10.1186/1471-2164-15-704 (PMC4150953; doi:10.1186/1471-2164-15-704)
Supplement: Supplementary file 3 — Additional file 3: Table S2: Ribosomal orthologous transcripts present in the Sf_TR2012b assembly. We used a manual annotation of Bombyx mori ribosomal proteins (David Heckel, personal communication) to search the transcripts present in our assembly using the tblastn algorithm [49, 50]. The ribosomal protein names in the first column follow the nomenclature developed for the rat. The one exception is RpS11 that exists as two closely related paralogs in all Lepidoptera (even Drosophila). However, both BmRpS11-1 and BmRpS11-2 match with the same transcript in our transcriptome assembly: joint2_rep_c105. In this table, we present every match that has over 50% identity and over 50% coverage of the query sequence. blastx against nr have been performed and the best hit for each S. frugiperda transcript has been represented with its evalue. In the last column, we dubbed a S. frugiperda sequence full if it has over 80% coverage and over 80% aminoacids identity with the query sequence (first column). It is ‘partial’ if it has less than 80% coverage. (PDF 41 KB) [file 12864_2014_6384_MOESM3_ESM.pdf]

<table>
<tr><th>Drosophila Hox Domain Name</th><th>% identity</th><th>Sf_TR2012b ID</th><th>Size (nts)</th><th>Best Blastx</th><th>E-value</th></tr>
<tr><td>HDRp1842|Dll|Distal-less|Drosophila</td><td>100</td><td>c12466</td><td>1186</td><td>>gi|357608245|gb|EHJ65890,1|Distal-less Dll limb-patterning protein [Danaus plexippus]</td><td>2,00E-124</td></tr>
<tr><td>HDRp1805|Awh|Arrowhead|Drosophila</td><td>46.3</td><td rowspan="2">c17432</td><td rowspan="2">421</td><td rowspan="2">>gi|512934125|ref|XP_004932958,1|PREDICTED: zinc finger homeobox protein 3-like [Bombyx mori]</td><td rowspan="2">2,00E-60</td></tr>
<tr><td>HDRp1789|onecut|onecut|Drosophila</td><td>32.69</td></tr>
<tr><td>HDRp1768|tup|tailup|Drosophila</td><td>96</td><td>c20198</td><td>975</td><td>>gi|512898223|ref|XP_004924347,1|PREDICTED: insulin gene enhancer protein ISL-1-like [Bombyx mori]</td><td>4,00E-52</td></tr>
<tr><td>HDRp1807|ara|araucan|Drosophila</td><td>100</td><td rowspan="5">c21575</td><td rowspan="5">936</td><td rowspan="5">>gi|512921196|ref|XP_004929820,1|PREDICTED: homeobox protein araucan-like [Bombyx mori]</td><td rowspan="5">1,00E-135</td></tr>
<tr><td>HDRp1817|caup|caupolican|Drosophila</td><td>98.33</td></tr>
<tr><td>HDRp528|mirr|mirror|Drosophila</td><td>96.67</td></tr>
<tr><td>HDRp1765|achi|achintya|Drosophila</td><td>45.16</td></tr>
<tr><td>HDRp1797|vis|vismay|Drosophila</td><td>45.16</td></tr>
<tr><td>HDRp1787|zfh1|Zn finger homeodomain 1|Drosophila</td><td>69.05</td><td>c24034</td><td>697</td><td>>gi|357614270|gb|EHJ68998,1|putative zinc finger protein [Danaus plexippus]</td><td>9,00E-84</td></tr>
<tr><td>HDRp1808|dve|defective proventriculus|Drosophila</td><td>42.31</td><td>c25458</td><td>1022</td><td>>gi|357627828|gb|EHJ77379,1|hypothetical protein KGM_06737 [Danaus plexippus]</td><td>9,00E-30</td></tr>
<tr><td>HDRp1830|repo|reversed polarity|Drosophila</td><td>96.08</td><td rowspan="8">c25575</td><td rowspan="8">735</td><td rowspan="8">>gi|357626860|gb|EHJ76772,1|hypothetical protein KGM_02204 [Danaus plexippus]</td><td rowspan="8">3,00E-78</td></tr>
<tr><td>HDRp1825|Rx|Retinal Homeobox|Drosophila</td><td>74.51</td></tr>
<tr><td>HDRp1791|hbn|homeobrain|Drosophila</td><td>74.51</td></tr>
<tr><td>HDRp1784|CG33980|CG33980|Drosophila</td><td>70.59</td></tr>
<tr><td>HDRp1831|unc-4|unc-4|Drosophila</td><td>70.59</td></tr>
<tr><td>HDRp1843|CG4136|CG4136|Drosophila</td><td>69.39</td></tr>
<tr><td>HDRp1832|OdsH|Ods-site homeobox|Drosophila</td><td>64.71</td></tr>
<tr><td>HDRp1827|Gsc|Goosecoid|Drosophila</td><td>63.27</td></tr>
<tr><td>HDRp426|al|aristaless|Drosophila</td><td>92</td><td rowspan="2">c27936</td><td rowspan="2">644</td><td rowspan="2">>gi|357614287|gb|EHJ69003,1|paired-like family homeodomain transcription factor [Danaus plexippus]</td><td rowspan="2">9,00E-37</td></tr>
<tr><td>HDRp1780|Pph13|PvuII-PstI homology 13|Drosophila</td><td>78</td></tr>
<tr><td>HDRp1812|pb|proboscipedia|Drosophila</td><td>97.67</td><td>c29970</td><td>330</td><td>>gi|167234210|ref|NP_001107807,1|maxillopedia [Tribolium castaneum]</td><td>3,00E-36</td></tr>
<tr><td>HDRp1811|inv|invected|Drosophila</td><td>76.92</td><td>c31882</td><td>455</td><td>>gi|223268546|emb|CAX36786,1|engrailed protein [Papilio dardanus]</td><td>2,00E-30</td></tr>
<tr><td>HDRp1809|vvl||Drosophila</td><td>89.58</td><td rowspan="5">c40494</td><td rowspan="5">412</td><td rowspan="5">>gi|195588294|ref|XP_002083893,1|GD13969 [Drosophila simulans]</td><td rowspan="5">1,00E-43</td></tr>
<tr><td>HDRp1792|nub|nubbin|Drosophila</td><td>55.1</td></tr>
<tr><td>HDRp1804|acj6|abnormal chemosensory jump 6|Drosophila</td><td>44</td></tr>
<tr><td>HDRp1826|pdm2|POU domain protein 2|Drosophila</td><td>42.86</td></tr>
<tr><td>HDRp1799|pdm3|pou domain protein 3|Drosophila</td><td>40.38</td></tr>
<tr><td>HDRp434|exd|extradenticle|Drosophila</td><td>100</td><td>c9983</td><td>1015</td><td>>gi|512916571|ref|XP_004928672,1|PREDICTED: homeobox protein extradenticle isoform X3 [Bombyx mori]</td><td>1,00E-59</td></tr>
<tr><td>HDRp440|eve|even-skipped|Drosophila</td><td>57.63</td><td>joint2_c3846</td><td>860</td><td>>gi|512924353|ref|XP_004930574,1|PREDICTED: homeobox protein Hox-D3-like [Bombyx mori]</td><td>5,00E-19</td></tr>
<tr><td>HDRp1764|CG7056|CG7056|Drosophila</td><td>75.86</td><td rowspan="2">joint2_c4772</td><td rowspan="2">907</td><td rowspan="2">>gi|357624439|gb|EHJ75221,1|hypothetical protein KGM_13906 [Danaus plexippus]</td><td rowspan="2">1,00E-37</td></tr>
<tr><td>HDRp1781|Six4|Six4|Drosophila</td><td>35.42</td></tr>
<tr><td>HDRp1806|Lim1||Drosophila</td><td>93.33</td><td rowspan="6">joint2_c6386</td><td rowspan="6">1450</td><td rowspan="6">>gi|512917506|ref|XP_004928903,1|PREDICTED: LIM/homeobox protein Lhx1-like [Bombyx mori]</td><td rowspan="6">0,00E+00</td></tr>
<tr><td>HDRp1761|Lim3|Lim3|Drosophila</td><td>70.18</td></tr>
<tr><td>HDRp1798|CG32105|CG32105|Drosophila</td><td>50.88</td></tr>
<tr><td>HDRp1802|ap|apterous|Drosophila</td><td>49.09</td></tr>
<tr><td>HDRp1793|CG4328|CG4328|Drosophila</td><td>47.37</td></tr>
<tr><td>HDRp1783|zfh2|Zn finger homeodomain 2|Drosophila</td><td>35.19</td></tr>
<tr><td>HDRp1763|unpg|unplugged|Drosophila</td><td>94.23</td><td>joint2_c6864</td><td>523</td><td>>gi|389614519|dbj|BAM20307,1|homeobox protein unplugged, partial [Papilio xuthus]</td><td>6,00E-29</td></tr>
<tr><td>HDRp1769|gsb|gooseberry|Drosophila</td><td>88.33</td><td rowspan="13">joint2_c7036</td><td rowspan="13">1228</td><td rowspan="13">>gi|512929372|ref|XP_004931804,1|PREDICTED: protein gooseberry-like [Bombyx mori]</td><td rowspan="13">4,00E-127</td></tr>
<tr><td>HDRp1796|gsb-n|gooseberry-neuro|Drosophila</td><td>86.44</td></tr>
<tr><td>HDRp500|prd|paired|Drosophila</td><td>83.05</td></tr>
<tr><td>HDRp1816|CG34340||Drosophila</td><td>70.69</td></tr>
<tr><td>HDRp1841|PHDP|Putative homeodomain protein|Drosophila</td><td>67.8</td></tr>
<tr><td>HDRp1766|CG9876|CG9876|Drosophila</td><td>66.67</td></tr>
<tr><td>HDRp1777|CG32532|CG32532|Drosophila</td><td>64.41</td></tr>
<tr><td>HDRp1773|CG34367||Drosophila</td><td>64.41</td></tr>
<tr><td>HDRp1803|eyg|eyegone|Drosophila</td><td>62.07</td></tr>
<tr><td>HDRp1818|toe|twin of eyg|Drosophila</td><td>62.07</td></tr>
<tr><td>HDRp1790|Ptx1|Ptx1|Drosophila</td><td>61.02</td></tr>
<tr><td>HDRp1837|oc|ocelliless|Drosophila</td><td>60</td></tr>
<tr><td>HDRp491|ey||Drosophila</td><td>59.65</td></tr>
</table>

HDRp1778|toy|twin of eyeless|Drosophila	59.65				
HDRp1782|ct|cut|Drosophila	40.68				
HDRp1833|lbe|ladybird early|Drosophila	95	joint2_c8241	1012	>gi|512934804|ref|XP_004933121,1|PREDICTED: transcription factor LBX1-like [Bombyx mori]	1,00E-88
HDRp1823|lbl|ladybird late|Drosophila	95				
HDRp1814|B-H2|BarH2|Drosophila	62.07				
HDRp1788|B-H1|BarH1|Drosophila	60.34				
HDRp1815|CG11617|CG11617|Drosophila	74.29	joint2_c9026	867	>gi|357611643|gb|EHJ67580,1|hypothetical protein KGM_02032 [Danaus plexippus]	4,00E-61
HDRp1795|Lag1|Longevity assurance|Drosophila	58.82	joint2_c9378	2272	>gi|512921866|ref|XP_004929979,1|PREDICTED: probable serine/threonine-protein kinase tsuA-like [Bombyx mori]	6,00E-115
HDRp481|lab|labial|Drosophila	91.38	joint2_rep_c1029	2583	>gi|357605492|gb|EHJ64645,1|hypothetical protein KGM_10843 [Danaus plexippus]	1,00E-68
HDRp1840|cad|caudal|Drosophila	84.75	joint2_rep_c611	799	>gi|52693900|dbj|BAD51739,1|caudal [Gryllus bimaculatus]	3,00E-29
HDRp1775|hth|homothorax|Drosophila	98.08	joint2_rep_c7818	896	>gi|576938426|dbj|BAO48186,1|homothorax [Bombyx mori]	1,00E-96
HDRp503|so|sine oculis|Drosophila	42.86				
HDRp1776|Optix|Optix|Drosophila	37.04				
HDRp1820|otp|orthopedia|Drosophila	100	L13602_T1	220	>gi|319891032|gb|ADV76083,1|orthopedia [Biston betularia]	1,00E-29
HDRp1800|CG11294|CG11294|Drosophila	68.09				
HDRp436|bap||Drosophila	88.33	L17218_T1	326	>gi|512934812|ref|XP_004933123,1|PREDICTED: homeobox protein bagpipe-like [Bombyx mori]	2,00E-46
HDRp512|vnd||Drosophila	68.33				
HDRp1771|Hmx||Drosophila	66.1				
HDRp1834|scro|scarecrow|Drosophila	66.1				
HDRp513|tin|tinman|Drosophila	60.34				
HDRp1844|CG12361|CG12361|Drosophila	58.82				
HDRp1838|CG11085|CG11085|Drosophila	56.9				
HDRp1770|bsh|brain-specific homeobox|Drosophila	55.93				
HDRp1821|H2,0|Homeodomain protein 2,0|Drosophila	55.77				
HDRp1794|C15|C15|Drosophila	55.17				
HDRp1828|HGTX|HGTX|Drosophila	52.54				
HDRp1785|CG14578|CG14578|Drosophila	50				
HDRp1370|bcd|bicoid|Drosophila	50				
HDRp1822|Dr|Drop|Drosophila	81.03	L330_T3	755	>gi|357603008|gb|EHJ63592,1|hypothetical protein KGM_02241 [Danaus plexippus]	4,00E-24
HDRp1836|CG15696|CG15696|Drosophila	53.45				
HDRp1835|CG34031|CG34031|Drosophila	53.33				
HDRp441|Dfd|Deformed|Drosophila	100	L6902_T1	534	>gi|112983614|ref|NP_001037341,1|transcription factor deformed [Bombyx mori]	3,00E-107
HDRp435|ftz|fushi-tarazu|Drosophila	84.78				
HDRp1813|Scr|Sex combs reduced|Drosophila	83.33				
HDRp427|Antp|Antennapedia|Drosophila	83.05				
HDRp1829|Ubx|Ultrabithorax|Drosophila	82.61				
HDRp511|Ubx||Drosophila	82.61				
HDRp1839|abd-A|abdominal A|Drosophila	77.59				
HDRp510|zen|zercknult|Drosophila	68.97				
HDRp1810|ind|intermediate neuroblasts defective|Drosophila	67.24				
HDRp1801|zen2|zerknullt-related|Drosophila	63.33				
HDRp1819|exex|extra-extra|Drosophila	62.07				
HDRp1767|ro|rough|Drosophila	60				
HDRp1760|btn|buttonless|Drosophila	57.89				
HDRp452|en|engrailed|Drosophila	55.17				
HDRp1762|Abd-B|Abdominal-B|Drosophila	54.24				
HDRp1772|E5|E5|Drosophila	61.4	rep_c11157	497	>gi|512935737|ref|XP_004933347,1|PREDICTED: barH-like 1 homeobox protein-like [Bombyx mori]	3,00E-59
HDRp1824|ems|empty spiracles|Drosophila	59.65				
HDRp1779|NK7,1|NK7,1|Drosophila	57.89				
HDRp1845|CG18599|CG18599|Drosophila	50				
HDRp1774|CG13424|CG13424|Drosophila	79.66	rep_c5853	511	>gi|512935737|ref|XP_004933347,1|PREDICTED: barH-like 1 homeobox protein-like [Bombyx mori]	7,00E-51
HDRp1786|slou|slouch|Drosophila	63.79				
HDRp501|pros|prospero|Drosophila	100	rep_c9681	1206	>gi|357619048|gb|EHJ71784,1|hypothetical protein KGM_10139 [Danaus plexippus]	3,00E-104
