# Supplementary material for: Establishment and analysis of a reference transcriptome for Spodoptera frugiperda
Source: BMC Genomics. 2014 Aug 23;15(1):704. doi: 10.1186/1471-2164-15-704 (PMC4150953; doi:10.1186/1471-2164-15-704)
Supplement: Supplementary file 4 — Additional file 4: Table S3: Homeodomain protein orthologs in Sf_TR2012b. This table shows the tblastn results using 108 Drosophila homeodomain sequences from the Homeodomain Resource Database [9]. The legend is as in Additional file 3: Table S2 except that only the best Sf_TR2012b match for each Hox-domain sequence has been reported and coverage value have been omitted since we searched for a small domain only. For each of the S. frugiperda transcripts detected, a blastx of the transcript sequence against the nr database has been performed and the best hit has been reported in the table as well as the e-value of the hit. (PDF 57 KB) [file 12864_2014_6384_MOESM4_ESM.pdf]

Gene name	Sf_TR2012b ID	Size (nt)	% identity between orthologs	Signal peptide	Best Blastx	E-value	Status
**General, Odorant and Pheromone Binding Proteins**							
GOBP1	c7994	899	65.0	N	>gi|121485827|gb|ABM54823.1| general odorant-binding protein GOBP1 [Spodoptera litura]	8E-69	Partial
GOBP2	joint2_rep_c3475	650	90.7	Y	>gi|10129671|emb|CAC08211.1| general odorant-binding protein 2 precursor (GOBP2) [Helicoverpa armigera]	1E-108	Full
OBP1	rep_c2034	694	47.2	Y	>gi|524902670|gb|AGR39564.1| odorant binding protein 1, partial [Agrotis ipsilon]	8E-71	Partial
OBP2	joint2_rep_c9255	1387	98.4	Y	>gi|389608671|dbj|BAM17945.1| unknown secreted protein [Papilio xuthus]	3E-113	Full
OBP3	rep_c490	1156	87.6	Y	>gi|324103929|gb|ADY17884.1| odorant binding protein [Spodoptera exigua]	6E-56	Full
OBP4	rep_c877	808	94.0	Y	>gi|469664301|gb|AGH70105.1| odorant binding protein 9 [Spodoptera exigua]	2E-85	Full
OBP5	rep_c2055	649	97.5	Y	>gi|324103925|gb|ADY17882.1| odorant binding protein [Spodoptera exigua]	1E-105	Full
OBP6	c15965	578	33.3	N	>gi|528321513|gb|AGS36743.1| OBP1 [Sesamia inferens]	2E-54	Partial
OBP7	rep_c571	524	98.6	Y	>gi|469664299|gb|AGH70104.1| odorant binding protein 8 [Spodoptera exigua]	1E-93	Full
OBP8	joint2_rep_c7287	1361	92.9	Y	>gi|290965852|gb|ADD71058.1| odorant-binding protein [Chilo suppressalis]	6E-116	Full
OBP9	Not found						
OBP10	c11418	845	64.9	Y	>gi|469664305|gb|AGH70107.1| odorant binding protein 11 [Spodoptera exigua]	2E-101	Full
OBP11	joint2_rep_c104	710	89.1	Y	>gi|39579195|gb|AAR28762.1| odorant-binding protein [Spodoptera frugiperda]	7E-87	Full
OBP12	rep_c504	652	87.2	Y	>gi|324103923|gb|ADY17881.1| antennal binding protein [Spodoptera exigua]	8E-80	Full
OBP13	c32728	702	44.9	N	>gi|443429763|gb|AGC92793.1| odorant-binding protein 19 [Helicoverpa assulta]	1E-55	Partial
OBP14	joint2_c2406	680	74.5	Y	>gi|469664297|gb|AGH70103.1| odorant binding protein 7 [Spodoptera exigua]	1E-73	Full
OBP15	joint2_rep_c3798	512	38.2	N	>gi|519767939|gb|AGP03458.1| SexiOBP12 [Spodoptera exigua]	8E-29	Partial
OBP16	Not found						
OBP17	rep_c44968	502	73.4	N	>gi|383215092|gb|AFG72998.1| odorant-binding protein 1 [Cnaphalocrocis medinalis]	2E-74	Partial
OBP18	rep_c2988	604	85.3	Y	>gi|328879864|gb|AEB54589.1| OBP8 [Helicoverpa armigera]	1E-86	Full
OBP19	Not found						
OBP20	Not found						
OBP22	joint2_rep_c1191	663	57.1	N	>gi|519767930|gb|AGP03456.1| SexiOBP10 [Spodoptera exigua]	3E-85	Partial
OBP23	rep_c3716	858	81.1	Y	>gi|39579207|gb|AAR28763.1| odorant-binding protein-2 precursor [Spodoptera frugiperda]	5E-79	Full
OBP24	joint2_c2045	719	70.5	N	>gi|39579195|gb|AAR28762.1| odorant-binding protein [Spodoptera frugiperda]	1E-55	Partial
OBP25	joint2_c2317	540	63.3	Y	>gi|324103931|gb|ADY17885.1| odorant binding protein [Spodoptera exigua]	4E-63	Full
OBP26	rep_c2960	663	87.0	Y	>gi|519767933|gb|AGP03457.1| SexiOBP11 [Spodoptera exigua]	1E-94	Full
OBP27	c28274	621	57.9	N	>gi|519767927|gb|AGP03455.1| SexiOBP9 [Spodoptera exigua]	2E-45	Partial
OBP28	joint2_c6322	583	65.3	N	>gi|519767945|gb|AGP03460.1| SexiOBP14 [Spodoptera exigua]	2E-73	Partial
OBP29	joint2_rep_c3596	552	79.7	N	>gi|324103933|gb|ADY17886.1| odorant binding protein [Spodoptera exigua]	1E-91	Partial
OBP30	rep_c4239	666	54.7	N	>gi|392522236|gb|AFM77984.1| odorant binding protein 6 [Spodoptera exigua]	6E-82	Partial
OBP31	Not found						
PBP1	joint2_c4216	1230	86.1	Y	>gi|43439928|gb|AAS46620.1| pheromone binding protein 1 [Spodoptera exigua]	3E-72	Full
PBP2	rep_c1860	787	95.7	Y	>gi|72169224|gb|AAS55551.2| pheromone binding protein 2 [Spodoptera exigua]	3E-91	Full
PBP3	rep_c5672	556	90.2	Y	>gi|266706212|gb|ACY78413.1| pheromone binding protein 3 [Spodoptera exigua]	8E-95	Full
No ortholog	rep_c4733	645		Y	>gi|519767924|gb|AGP03454.1| SexiOBP8 [Spodoptera exigua]	2E-30	Partial
No ortholog	rep_c7113	608		Y	>gi|519767924|gb|AGP03454.1| SexiOBP8 [Spodoptera exigua]	5E-40	Full
No ortholog	c17007	503		Y	>gi|519767924|gb|AGP03454.1| SexiOBP8 [Spodoptera exigua]	2E-18	Partial
No ortholog	c18292	449		Y	>gi|443429755|gb|AGC92789.1| odorant-binding protein 9 [Helicoverpa assulta]	1E-16	Partial
No ortholog	joint2_rep_c1590	628		N	>gi|519767924|gb|AGP03454.1| SexiOBP8 [Spodoptera exigua]	5E-09	Partial
No ortholog	joint2_rep_c2573	691		Y	>gi|328879848|gb|AEB54581.1| OBP5 [Helicoverpa armigera]	1E-47	Full
No ortholog	joint2_c5045	2731		N	>gi|512924812|ref|XP_004930684.1| PREDICTED: uncharacterized protein LOC101740799 [Bombyx mori]	9E-156	Partial
**Chemosensory Proteins**							
CSP1	joint2_rep_c612	786	89.8	Y	>gi|552955230|gb|AGY49269.1| putative chemosensory protein [Sesamia inferens]	5E-61	Full
CSP2	joint2_rep_c3099	647	99.2	Y	>gi|365919036|gb|AEX07265.1| CSP2 [Helicoverpa armigera]	4E-73	Full
CSP3	Not found						

CSP4	joint2_c3581	768	79.1	N	>gi|552955232|gb|AGY49270.1| putative chemosensory protein [Sesamia inferens]	1E-68	Partial
CSP5	rep_c3130	873	95.3	Y	>gi|122894084|gb|ABM67688.1| chemosensory protein CSP1 [Spodoptera exigua]	2E-75	Full
CSP6	joint2_rep_c2279	479	97.6	Y	>gi|524903217|gb|AGR39578.1| chemosensory protein 8 [Agrotis ipsilon]	1E-76	Full
CSP7	joint2_c1683	991	92.5	Y	>gi|524903124|gb|AGR39575.1| chemosensory protein 5 [Agrotis ipsilon]	2E-61	Full
CSP8	rep_c4613	664	92.2	Y	>gi|122894086|gb|ABM67689.1| chemosensory protein CSP2 [Spodoptera exigua]	3E-67	Full
CSP9	rep_c23815	508	73.8	Y	>gi|63020522|gb|AAY26143.1| chemosensory protein CSP [Spodoptera litura]	3E-48	Full
CSP10	c10036	724	53.4	Y	>gi|112983038|ref|NP_001037069.1| chemosensory protein 9 precursor [Bombyx mori]	1E-74	Full
CSP11	joint2_c9626	672	64.7	Y	>gi|13236828|gb|AAK14793.1| sensory appendage protein-like protein [Mamestra brassicae]	5E-34	Full
CSP12	joint2_c1394	1032	86.3	Y	>gi|156616773|gb|ABU87405.1| chemosensory-like protein [Trichoplusia ni]	4E-31	Full
CSP13	rep_c3399	674	98.4	Y	>gi|365919040|gb|AEX07267.1| CSP6 [Helicoverpa armigera]	2E-60	Full
CSP14	joint2_c3078	633	88.3	Y	>gi|524903160|gb|AGR39576.1| chemosensory protein 6 [Agrotis ipsilon]	3E-50	Full
CSP15	rep_c4149	691	81.0	Y	>gi|148298809|ref|NP_001091781.1| chemosensory protein 15 [Bombyx mori]	5E-21	Full
CSP16	joint2_c2765	609	56.6	Y	>gi|461726423|gb|AGH20054.1| chemosensory protein 16, partial [Helicoverpa armigera]	7E-32	Full
CSP17	rep_c28057	364	87.7	Y	>gi|207107800|dbj|BAG71921.1| chemosensory protein 13 [Papilio xuthus]	8E-38	Full
CSP17	rep_c475	987	87.7	Y	>gi|207107800|dbj|BAG71921.1| chemosensory protein 13 [Papilio xuthus]	1E-55	Full
CSP18	joint2_c9032	547	79.3	Y	>gi|357621531|gb|EHJ73331.1| chemosensory protein 12 [Danaus plexippus]	5E-37	Full
CSP19	rep_c601	1264	90.2	Y	>gi|524903017|gb|AGR39572.1| chemosensory protein 2 [Agrotis ipsilon]	1E-62	Full
CSP20	rep_c14062	454	86.2	Y	>gi|405117276|gb|AFR92094.1| chemosensory protein 10 [Helicoverpa armigera]	2E-56	Partial
No ortholog	c35762	396		Y	>gi|357611229|gb|EHJ67380.1| chemosensory protein [Danaus plexippus]	2E-12	Partial
No ortholog	rep_c44394	568		Y	>gi|122894084|gb|ABM67688.1| chemosensory protein CSP1 [Spodoptera exigua]	2E-30	Partial
No ortholog	rep_c51916	628		Y	>gi|122894086|gb|ABM67689.1| chemosensory protein CSP2 [Spodoptera exigua]	6E-42	Partial
**Olfactory receptors**				**Frameshift**			
SlitOR13	c13744	1272	NA	Y	>gb|AEY84942.2| putative olfactory receptor 1 [Spodoptera litura]	1E-154	NA
SlitOR6	c24282	422	NA	Y	>gb|AGI96748.1| olfactory receptor 6 [Spodoptera litura]	2E-13	NA
SlitOR16	c38866	360	NA	STOP codon	>gb|ACL81182.1| putative olfactory receptor 16 [Spodoptera littoralis]	2E-44	NA
SlitOR17	c29382	571	NA	Y	>emb|CAG38118.1| putative chemosensory receptor 17 [Heliothis virescens]	2E-25	NA
SlitOR41	c36553	622	NA	Y	>gb|AGK90009.1| olfactory receptor 20 [Helicoverpa armigera]	6E-56	NA
SlitOR31	c38188	371	NA	Y	>gb|AGY14586.1| putative odorant receptor, partial [Sesamia inferens]	1E-43	NA
SlitOR25	c41185	470	NA	Y	>ref|NP_001166621.1| olfactory receptor 64 [Bombyx mori]	7E-22	NA
SlitOR28	c42933	592	NA	Y +STOP codon	>gb|AGG08878.1| putative olfactory receptor 12 [Spodoptera litura]	4E-75	NA
SlitOR29	c42948	593	NA	Y +STOP codon	>gb|AGY14590.1| putative odorant receptor, partial [Sesamia inferens]	5E-31	NA
SlitOR3	L10788_T1	221	NA	N	>gb|AEF32141.1| odorant receptor [Spodoptera exigua]	2E-21	NA
SlitOR2 (ORco)	joint2_c3978	1130	NA	Y	>gb|AAW52583.1| putative chemosensory receptor 2 [Spodoptera exigua]	0E+00	NA
**Ionotropic receptors**				**Frameshift**			
SlitIR25a	c34962	409	NA	N	>ref|XP_004927662.1| PREDICTED: uncharacterized protein LOC101735556 [Bombyx mori]	3E-54	NA
SlitIR25a	c15409	635	NA	N	>gb|ADR64679.1| putative chemosensory ionotropic receptor IR25a [Spodoptera littoralis]	5E-38	NA
SlitIR8a	c42892	374	NA	N	>ref|XP_004932768.1| PREDICTED: uncharacterized protein LOC101738187 [Bombyx mori]	4E-52	NA
SlitIR41a	c36556	367	NA	N	>gb|ADR64681.1| putative chemosensory ionotropic receptor IR41a [Spodoptera littoralis]	5E-12	NA
No Slit ortholog	c26803	708	NA	N	>ref|XP_004927609.1| PREDICTED: glutamate receptor ionotropic, kainate 3-like [Bombyx mori]	2E-12	NA
