# Supplementary material for: Establishment and analysis of a reference transcriptome for Spodoptera frugiperda
Source: BMC Genomics. 2014 Aug 23;15(1):704. doi: 10.1186/1471-2164-15-704 (PMC4150953; doi:10.1186/1471-2164-15-704)
Supplement: Supplementary file 5 — Additional file 5: Table S4: Genes of olfaction. Candidate odorant-binding protein (OBP) and chemosensory protein (CSP) encoding genes identified in Sf_TR2012b by tblastn using 57 proteins annotated in the closely related species S. littoralis [10–12]. The occurrence of a signal peptide, a hallmark for OBPs and CSPs, is indicated (Y, yes; N, no). Full-length coding sequences are also indicated. Candidate olfactory receptor (OR) and ionotropic receptor (IR) encoding genes identified in Sf_TR2012b by tblastn using 64 proteins annotated in the closely related species S. littoralis [10–12]. Identified frameshifts or unexpected stop codons in the coding sequences are indicated (Y, yes; N, no). (PDF 171 KB) [file 12864_2014_6384_MOESM5_ESM.pdf]

Gene name	Sf_TR2012b ID	Size (nt)	Best Blastx	E-value	Status
**GNBP**					
	joint2_c3183	1616	>gi|326537522|gb|ADZ93428.1| beta-1,3-glucan binding protein [*Spodoptera litura*]	0E+00	Full
	joint2_rep_c3016	1215	>gi|350627242|gb|AEQ33590.1| beta-1,3-glucan recognition protein 2 [*Spodoptera litura*]	2E-75	Partial
	joint2_c4285	653	>gi|208972529|gb|ACI32825.1| beta-1,3-glucan recognition protein 1 [*Helicoverpa armigera*]	2E-88	Partial
**PGRP**					
	c10154	2185	>gi|357631551|gb|EHJ79021.1| peptidoglycan recognition protein-lc [*Danaus plexippus*]	9E-37	Full
	joint2_rep_c2027	1444	>gi|18202160|sp|O76537.1|PGRP_TRINI RecName: Full=Peptidoglycan recognition protein	2E-96	Full
	rep_c24829	1270	>gi|18202160|sp|O76537.1|PGRP_TRINI RecName: Full=Peptidoglycan recognition protein	4E-98	Full
	c6812	1214	>gi|399128031|gb|AFP23116.1| peptidoglycan recognition protein B [*Helicoverpa armigera*]	8E-105	Full
	c18686	904		6E-51	Full
	rep_c2782	2079	>gi|399128033|gb|AFP23117.1| peptidoglycan recognition protein C [*Helicoverpa armigera*]	1E-91	Full
	rep_c7951	1190	>gi|112982725|ref|NP_001037560.1| peptidoglycan recognition protein S2 precursor [*Bombyx mori*]	2E-51	Full
	joint2_c7700	1582	>gi|357631551|gb|EHJ79021.1| peptidoglycan recognition protein-lc [*Danaus plexippus*]	1E-34	Partial
	L34141_T1	107	>gi|315507103|gb|ADU33187.1| peptidoglycan recognition protein D [*Ostrinia nubilalis*]	1E-14	Partial
	c21899	572	>gi|357612138|gb|EHJ67829.1| hypothetical protein KGM_02561 [*Danaus plexippus*]	3E-30	Partial
	c18209	545	>gi|315507103|gb|ADU33187.1| peptidoglycan recognition protein D [*Ostrinia nubilalis*]	8E-17	Partial
**TEP (Thioester cont. protein)**					
	c41436	333	>gi|357605113|gb|EHJ64472.1| putative tep3 [*Danaus plexippus*]	2E-43	Partial
**Transmembrane receptors**					
P77	c28367	444	>gi|305632939|dbj|BAJ16182.1| p77 homologue [*Spodoptera litura*]	2E-38	Partial
Eater (EGF-domain protein)	joint2_c4820	1854	>gi|300440347|gb|ADK20108.1| eater [*Drosophila melanogaster*]	8E-26	Partial
	rep_c13922	1823		4E-29	
	joint2_c5622	2280		4E-68	
	joint2_rep_c6592	1709		2E-44	
DSCAM	c28502	372	>gi|357628590|gb|EHJ77866.1| dscam [*Danaus plexippus*]	5E-31	Partial
	c31413	429		9E-57	
	c36605	482		3E-46	
	c37546	1130		1E-149	
Nimrod	joint2_rep_c2173	1968	>gi|112983550|ref|NP_001036879.1| nimrod B precursor [*Bombyx mori*]	4E-66	Partial
	joint2_rep_c8963	2140		1E-113	
Scavenger receptors	joint2_c2139	2191	>gi|283945476|ref|NP_001164650.1| scavenger receptor class B member 3 [*Bombyx mori*]	7E-41	Partial
	c34896	803	>gi|283483658|dbj|BAI66273.1| Cameo2 [*Bombyx mori*]	1E-92	Partial
	c15860	1244	>gi|283945476|ref|NP_001164650.1| scavenger receptor class B member 3 [*Bombyx mori*]	6E-89	Full
	rep_c5141	2229	>gi|82880638|gb|ABB92836.1| scavenger receptor SR-C-like protein [*Spodoptera frugiperda*]	0.0	Full
**Extra cellular signal transduction and cytokines**					
Persephone	c12701	731	>gi|56418393|gb|AAV91004.1| hemolymph proteinase 6 [*Manduca sexta*]	4E-41	Partial
	rep_c4857	1588		1E-155	
Grass-like	not found				
Spätzle-processing enzyme	joint2_rep_c2954	1774	>gi|357622297|gb|EHJ73832.1| hemolymph proteinase 8 [*Danaus plexippus*]	1E-114	Full
	L5381_T1	572		1E-58	Partial
Spätzle	joint2_rep_c4418	2173	>gi|388252721|gb|AFK24443.1| spatzle-2 [*Spodoptera frugiperda*]	2E-101	Full
Growth Blocking Peptide	rep_c48337	695	>gi|3986192|dbj|BAA34953.1| growth-blocking peptide [*Spodoptera litura*]	1E-46	Partial
	rep_c30358	535		5E-26	
	rep_c47928	464		7E-16	
	joint2_rep_c335	2634		7E-52	Full
Growth Blocking Peptide Binding Protein	c21183	1533	>gi|34392593|dbj|BAC82630.1| growth blocking peptide binding protein [*Mythimna separata*]	2E-98	Full
	joint2_rep_c3440	2125		1E-138	

hdd23	joint2_rep_c2127	625	>gi|4090970|gb|AAD09282.1| immune-related Hdd23 [*Hyphantria cunea*]	6E-15	Full
	rep_c50634	663		4E-14	
SRP	joint2_rep_c284	616	>gi|168701455|ref|ZP_02733732.1| hypothetical protein GobsU_18155 [*Gemmata obscuriglobus* UQM 2246]	5E-15	Full
	joint2_rep_c3270	584	>gi|4090970|gb|AAD09282.1| immune-related Hdd23 [*Hyphantria cunea*]	1E-13	
HCP	joint2_rep_c520	831	>gi|357604127|gb|EHJ64054.1| mitochondria-associated granulocyte macrophage CSF signaling molecule [Danaus plexippus]	3E-49	Full
Upd3	not found				

Gene family	Sf_TR2012b ID	Size (nt)	Best Blastx	E-value	Status
**Toll pathway**					
Toll	c21297	983	>gi|357626027|gb|EHJ76269.1| putative protein toll precursor [*Danaus plexippus*]	1E-101	Partial
	c15374	651	>gi|357613547|gb|EHJ68577.1| putative toll [*Danaus plexippus*]	1E-63	
	c18222	564	>gi|357604744|gb|EHJ64305.1| putative toll [*Danaus plexippus*]	1E-84	
	c27553	438	>gi|357611178|gb|EHJ67353.1| putative toll [*Danaus plexippus*]	8E-52	
	c44947	511	>gi|357607081|gb|EHJ65350.1| putative toll [*Danaus plexippus*]	4E-81	
	c45159	661	>gi|357617307|gb|EHJ70712.1| putative toll [*Danaus plexippus*]	2E-71	
	c9825	1044	>gi|357613547|gb|EHJ68577.1| putative toll [*Danaus plexippus*]	2E-47	
	joint2_c10319	618	>gi|357613547|gb|EHJ68577.1| putative toll [*Danaus plexippus*]	2E-71	
	joint2_c10448	488	>gi|357617307|gb|EHJ70712.1| putative toll [*Danaus plexippus*]	4E-41	
	joint2_c7245	1199	>gi|357613547|gb|EHJ68577.1| putative toll [*Danaus plexippus*]	2E-97	
	joint2_c4264	345	>gi|357622857|gb|EHJ74227.1| putative toll-like receptor 13 [*Danaus plexippus*]	5E-38	
	joint2_c6344	799	>gi|357622857|gb|EHJ74227.1| putative toll-like receptor 13 [*Danaus plexippus*]	2E-96	
	c20425	698	>gi|389609959|dbj|BAM18591.1| Toll-like receptor [*Papilio xuthus*]	4E-50	
	joint2_c6812	1731	>gi|182511220|ref|NP_001116821.1| 18 wheeler precursor [*Bombyx mori*]	0.0	
	c16695	982	>gi|318104931|gb|ADV41489.1| toll receptor 18 wheeler [*Spodoptera frugiperda*]	0.0	
	c16515	659	>gi|357617686|gb|EHJ70927.1| 18 wheeler [*Danaus plexippus*]	3E-84	
	c37507	390	>gi|357627953|gb|EHJ77461.1| 18 wheeler [*Danaus plexippus*]	4E-40	
	c28269	407	>gi|318104931|gb|ADV41489.1| toll receptor 18 wheeler [*Spodoptera frugiperda*]	3E-45	
	c26372	456	>gi|318104931|gb|ADV41489.1| toll receptor 18 wheeler [*Spodoptera frugiperda*]	2E-32	
	c23998	356	>gi|363497934|gb|AEW24430.1| toll [*Spodoptera exigua*]	2E-48	
	joint2_c3284	2124	>gi|345479829|ref|XP_001604871.2| PREDICTED: protein toll [*Nasonia vitripennis*]	2E-76	
	c46305	637	>gi|91076464|ref|XP_971999.1| PREDICTED: similar to toll [*Tribolium castaneum*]	2E-81	
	c21766	731	>gi|357617307|gb|EHJ70712.1| putative toll [*Danaus plexippus*]	1E-64	
	c44912	551	>gi|91076464|ref|XP_971999.1| PREDICTED: similar to toll [*Tribolium castaneum*]	4E-72	
MyD88	joint2_c5546	2525	>gi|357622568|gb|EHJ73995.1| putative Myeloid differentiation primary response protein MyD88 [*Danaus plexippus*]	2E-55	Full
	joint2_c8044	1454	>gi|388252723|gb|AFK24444.1| MyD88 [*Spodoptera frugiperda*]	1E-164	Partial
Tollip	c14199	1951	>gi|512891539|ref|XP_004922709.1| PREDICTED: toll-interacting protein-like [*Bombyx mori*]	2E-10	Partial
	c19207	624	>gi|91081565|ref|XP_975168.1| PREDICTED: similar to Toll-interacting protein [*Tribolium castaneum*]	6E-26	
Tube	joint2_c8555	2183	>gi|357617711|gb|EHJ70951.1| hypothetical protein KGM_14811 [*Danaus plexippus*]	1E-104	Full
Pellino	joint2_rep_c3710	3514	>gi|512886824|ref|XP_004921823.1| PREDICTED: protein pellino-like [*Bombyx mori*]	3E-133	Partial
Pelle	c36088	429	>gi|385048188|gb|AFI39855.1| pelle, partial [*Daphnia parvula*]	2E-31	Full
	c42147	785	>gi|512906233|ref|XP_004926145.1| PREDICTED: serine/threonine-protein kinase pelle-like [*Bombyx mori*]	4E-42	
Traf	joint2_c8521	444	>gi|379698888|ref|NP_001243915.1| TRAF6 [*Bombyx mori*]	5E-23	Partial
	L13084_T1	146		2E-16	
	L12209_T1	163		3E-16	
	L13118_T1	145		3E-19	
Ecsit	joint2_c9334	1445	>gi|170035114|ref|XP_001845416.1| ecsit [*Culex quinquefasciatus*]	1E-100	Full
Cactus	joint2_c6735	2183	>gi|289629214|ref|NP_001166191.1| cactus [*Bombyx mori*]	2E-83	Full
Dorsal	joint2_c10766	2417	>gi|357614722|gb|EHJ69235.1| embryonic polarity protein dorsal [*Danaus plexippus*]	1E-103	Partial
DIF	Not found				
**Imd pathway**					
IMD	c38668	592	>gi|388597545|gb|AFK75934.1| IMD-like protein [*Spodoptera exigua*]	2E-54	Partial

IMD	joint2_c6888	536	>gi|388597545|gb|AFK75934.1| IMD-like protein [*Spodoptera exigua*]	2E-16	Partial
FADD	c7443	1377	>gi|321400088|ref|NP_001189465.1| fadd [*Bombyx mori*]	7E-51	Full
Dredd	Not found				
Caspar	joint2_c495	785	>gi|357616510|gb|EHJ70237.1| Fas associated factor 1 [*Danaus plexippus*]	9E-125	Partial
	joint2_c6556	837		2E-131	Partial
TAK1	joint2_c2598	1201	>gi|357624974|gb|EHJ75547.1| putative Mitogen-activated protein kinase kinase kinase 7 [*Danaus plexippus*]	5E-162	Partial
TAB2	joint2_rep_c4196	1252	>gi|357623679|gb|EHJ74734.1| TAK1-associated binding protein 2 isoform A *[Danaus plexippus]*	9E-66	Partial
IAP2	c41058	409	>gi|321400074|ref|NP_001189458.1| inhibitor of apoptosis 2 [*Bombyx mori*]	8E-21	Partial
	c39309	300		4E-12	Partial
IKKβ (Ird5)	joint2_c6441	998	>gi|357624008|gb|EHJ74934.1| Inhibitor of nuclear factor kappa B kinase beta subunit [*Danaus plexippus*]	1E-114	Partial
	joint2_c9835	1349		6E-41	Partial
IKKγ (Kenny)	Not found				
UBcC (Effete)	joint2_rep_c1636	1810	>gi|357604946|gb|EHJ64394.1| effete [*Danaus plexippus*]	5E-99	Full
Uev1a	joint2_c554	1742	>gi|512928183|ref|XP_004931520.1| PREDICTED: ubiquitin-conjugating enzyme E2 variant 2-like [*Bombyx mori*]	1E-92	Full
Ubc13 (Bend)	joint2_rep_c3381	1608	>gi|114051115|ref|NP_001040393.1| ubiquitin conjugating enzyme E2 [*Bombyx mori*]	3E-86	Full
Relish	c6453	2060	>gi|346987771|gb|AEO51739.1| relish [*Helicoverpa armigera*]	2E-180	Partial
	joint2_c9990	707	>gi|363497936|gb|AEW24431.1| relish [*Spodoptera exigua*]	3E-108	Partial
	c13122	717	>gi|363497936|gb|AEW24431.1| relish [*Spodoptera exigua*]	8E-105	Partial
**Negative regulators of IMD pathway**					
Pirk	Not found				
POSH	L4001_T1	1041	>gi|345487941|ref|XP_001606578.2| PREDICTED: SH3 domain-containing RING finger protein 3-like [*Nasonia vitripennis*]	7E-82	Partial
DNR1	joint2_c5988	1549	>gi|357619816|gb|EHJ72245.1| putative myosin regulatory light chain interacting protein [*Danaus plexippus*]	7E-79	Full
CYLD	c31819	418	>gi|512926367|ref|XP_004931068.1| PREDICTED: ubiquitin carboxyl-terminal hydrolase CYLD-like [*Bombyx mori*]	3E-46	Partial
skpA	rep_c323	1050	>gi|114052370|ref|NP_001040518.1| S-phase kinase-associated protein [*Bombyx mori*]	2E-96	Full
Cullin	rep_c7665	1161	>gi|91081955|ref|XP_967420.1| PREDICTED: similar to cullin [*Tribolium castaneum*]	5E-119	Partial
Slimb	L1465_T1	951	>gi|157092624|gb|ABV22506.1| Slimb [*Danaus plexippus*]	0.0	Partial
sickie	joint2_rep_c10241	2079	>gi|512916921|ref|XP_004928759.1| PREDICTED: protein sickie-like [Bombyx mori]	0.0	Partial
Scrawny (dUSP36)	L9905_T1	263	>gi|512928201|ref|XP_004931523.1| PREDICTED: ubiquitin carboxyl-terminal hydrolase 36-like [*Bombyx mori*]	4E-50	Partial
Akirin	joint2_rep_c1441	923	>gi|379699008|ref|NP_001243977.1| akirin protein [Bombyx mori]	8E-63	Partial
	rep_c255	1787			Partial
**JAK/STAT pathway**					
Domless	joint2_c9929	686	>gi|261335947|emb|CBH09280.1| putative tyrosine phosphatase [*Heliconius melpomene*]	8E-84	Partial
	joint2_c8943	355		1E-31	Partial
JAK (hopscotch)	c28082	598	>gi|357607423|gb|EHJ65487.1| putative tyrosine-protein kinase jak2 [*Danaus plexippus*]	1E-92	Partial
	c27398	511		2E-42	Partial
STAT (umpaired)	c10638	896	>gi|17225564|gb|AAL37476.1|AF329946_1 signal transducer and activator of transcription [*Spodoptera frugiperda*]	1E-151	Partial
	joint2_c4458	1227		0.0	
	c35515	393		7E-51	
	L3562_T1	402		2E-33	
	L3562_T2	349		2E-33	
PIAS	c23834	806	>gi|385259116|gb|AFI55458.1| PIAS2 protein [*Bombyx mori*]	1E-111	Partial
SOCS	joint2_c4404	2267	>gi|357620333|gb|EHJ72563.1| suppressor of cytokine signaling 6 [*Danaus plexippus*]	1E-136	Full
**JNK pathway**					
Hem	c28696	682	>gi|357616343|gb|EHJ70140.1| putative membrane-associated protein gex-3 [*Danaus plexippus*]	1E-102	Partial

<table>
<tr><td rowspan="3">Hem</td><td>rep_c2497</td><td>1579</td><td rowspan="2">>gi|357616343|gb|EHJ70140.1| putative membrane-associated protein gex-3 [Danaus plexippus]</td><td>1E-88</td><td rowspan="3">Partial</td></tr>
<tr><td>c34937</td><td>304</td><td>7E-45</td></tr>
<tr><td>c28148</td><td>426</td><td>>gi|332017367|gb|EGI58109.1| Membrane-associated protein Hem [Acromyrmex echinatior]</td><td>5E-58</td></tr>
<tr><td>Basket</td><td>Not found</td><td></td><td></td><td></td><td></td></tr>
<tr><td rowspan="4">Fos (kayak)</td><td>c12189</td><td>531</td><td rowspan="3">>gi|357622228|gb|EHJ73792.1| kayak isoform A [Danaus plexippus]</td><td>4E-52</td><td rowspan="4">Partial</td></tr>
<tr><td>c19720</td><td>449</td><td>7E-17</td></tr>
<tr><td>c16092</td><td>479</td><td>4E-15</td></tr>
<tr><td>c12451</td><td>1453</td><td>>gi|380012628|ref|XP_003690381.1| PREDICTED: proto-oncogene c-Fos-like [Apis florea]</td><td>3E-44</td></tr>
<tr><td rowspan="2">Jun</td><td>joint2_rep_c2246</td><td>2541</td><td>>gi|388540204|gb|AFK64813.1| c-jun-like protein [Helicoverpa armigera]</td><td>1E-132</td><td>Full</td></tr>
<tr><td>L23568_T1</td><td>114</td><td>>gi|357613266|gb|EHJ68412.1| c-Jun NH2-terminal kinase [Danaus plexippus]</td><td>3E-13</td><td>Partial</td></tr>
</table>

Gene name	Sf_TR2012b ID	Size (nt)	Best Blastx	E-value	Status
**Phenoloxidase System**					
PPAEs	joint2_c2301	1481	>gi|56718388|gb|AAW24480.1| prophenol oxidase activating enzyme 1 [*Spodoptera litura*]	0.0	Full
	rep_c3743	1665		0.0	
	L5381_T1	572	>gi|112983934|ref|NP_001036844.1| BzArgOEtase precursor [*Bombyx mori*]	1E-69	Partial
	joint2_rep_c7331	666	>gi|56718390|gb|AAW24481.1| prophenol oxidase activating enzyme 3 [*Spodoptera litura*]	7E-82	Full
	joint2_rep_c7313	1521		1E-107	Full
PPO1	joint2_rep_c2737	1440	>gi|82880634|gb|ABB92834.1| prophenoloxidase subunit 1 [*Spodoptera frugiperda*]	0E+00	Partial
	joint2_c8944	2240			
PPO2	joint2_rep_c165	2619	>gi|82880636|gb|ABB92835.1| prophenoloxidase subunit 2 [*Spodoptera frugiperda*]	0E+00	Full
Phenoloxidase Inhibitor	rep_c3203	599	>gi|116833169|gb|ABK29497.1| phenoloxidase inhibitor protein [*Helicoverpa armigera*]	4E-06	Full
	rep_c7945	509		2E-07	
**Antimicrobial peptides**					
Attacin	joint2_c9574	1003	>gi|1703758|sp|P50725.1|ATTA_TRINI RecName: Full=Attacin-A; Flags: Precursor	1E-83	Full
	joint2_c6638	924		7E-83	
	rep_c30214	1028		2E-85	
	rep_c16033	1116		9E-87	
	rep_c9395	995		2E-87	
	rep_c2883	925		6E-82	
	joint2_c4106	1139	>gi|238915975|gb|ACR78454.1| attacin [*Heliothis virescens*]	1E-64	Full
	joint2_rep_c2387	1517		2E-75	
	rep_c33582	445	>gi|159193|gb|AAA29183.1| attacin E precursor [*Hyalophora cecropia*]	5E-27	Partial
Cecropin	rep_c6862	742	>gi|61661433|gb|AAX51304.1| cecropin [*Helicoverpa armigera*]	2E-16	Full
	joint2_rep_c2859	555		9E-17	
	rep_c20433	561	>gi|46396047|sp|Q9XZG9.1|CECA_SPOLT RecName: Full=Cecropin-A	2E-17	Full
	joint2_rep_c4163	457	>gi|313247970|gb|ADR51148.1| cecropin 3 [*Helicoverpa armigera*]	3E-08	Full
	rep_c485	997	>gi|112983176|ref|NP_001037031.1| cecropin CBM2 precursor [*Bombyx mori*]	6E-11	Full
	rep_c1453	915		4E-11	
	rep_c16188	933		1E-11	
	rep_c1132	803	>gi|112984238|ref|NP_001037460.1| cecropin-B precursor [*Bombyx mori*]	8E-11	Full
	rep_c12210	1205	>gi|157704367|gb|ABV68872.1| cecropin B [*Trichoplusia ni*]	5E-06	Full
	joint2_rep_c1821	547	>gi|61660433|gb|AAX51193.1| HacD [*Helicoverpa armigera*]	5E-25	Full
	joint2_rep_c2857	500		3E-22	
	joint2_rep_c1566	959	>gi|1705742|sp|P50721.1|CE3E_HYPCU RecName: Full=Hyphancin-3E	3E-16	Full
	joint2_rep_c3462	539		1E-17	
	joint2_rep_c4099	519		4E-16	
	rep_c49718	749		3E-17	
	rep_c22144	383		3E-18	
Defensin	joint2_rep_c1200	643	>gi|157704371|gb|ABV68874.1| cobatoxin-like protein [*Trichoplusia ni*]	1E-11	Full

Defensin	rep_c31235	505	>gi|33439724|gb|AAQ18900.1| cobatoxin long form B [*Spodoptera frugiperda*]	1E-22	Full
	rep_c18211	391	>gi|33439716|gb|AAQ18896.1| gallerimycin [*Spodoptera frugiperda*]	3E-37	Full
	joint2_rep_c352	915		1E-33	chimere
	joint2_rep_c1506	611	>gi|32394732|gb|AAM96925.1| defensin precursor [*Spodoptera frugiperda*]	2E-47	Full
	rep_c50766	546		9E-53	
Gloverin	rep_c11387	765	>gi|302347126|gb|ADL27731.1| gloverin [*Spodoptera exigua*]	9E-59	Full
	rep_c15702	683		1E-12	Partial
	rep_c24985	441		1E-11	
	rep_c396	872		1E-58	Full
	rep_c448	850		2E-92	Full
Lebocin	joint2_rep_c2864	848	>gi|237506881|gb|ACQ99193.1| proline-rich protein [*Galleria mellonella*]	9E-39	Full
	joint2_rep_c3089	679		3E-32	
	rep_c31645	829		5E-40	
	joint2_rep_c6464	886		3E-39	
	joint2_rep_c4195	737	>gi|57157818|dbj|BAD84189.1| lebocin-like protein [*Samia ricini*]	6E-26	
	joint2_rep_c874	1180	>gi|219958088|gb|ACL68098.1| pro-lebocin [*Manduca sexta*]	3E-34	
	rep_c1072	1133		2E-25	
	rep_c24419	601		6E-16	
Lys1	rep_c48748	934	>gi|206598481|gb|ACI16106.1| lysozyme [*Spodoptera litura*]	9E-97	Full
Lys2	joint2_rep_c2972	960	>gi|44887636|gb|AAS48094.1| lysozyme [*Pseudoplusia includens*]	6E-60	Full
Lys3	rep_c18992	845	>gi|29893332|gb|AAP03061.1| lysozyme [*Spodoptera exigua*]	6E-34	Full
LLP1	rep_c2131	804	>gi|145286562|gb|ABP52098.1| lysozyme-like protein 1 [*Antheraea mylitta*]	6E-58	Full
LLP2	rep_c4916	794	>gi|357624808|gb|EHJ75441.1| lysozyme-like protein 1 [*Danaus plexippus*]	2E-46	Full
Moricin	c34807	535	>gi|343915878|gb|AEM66431.1| moricin B3 [*Bombyx mandarina*]	2E-11	Full
	joint2_rep_c3441	403	>gi|146738004|gb|ABQ42580.1| moricin-like peptide D [*Galleria mellonella*]	1E-11	Full
	rep_c45075	365		7E-13	
	rep_c9540	383		6E-15	
Spod-x-tox	joint2_rep_c4528	740	>gi|379045801|gb|AFC87713.1| Spod-11-tox b protein [*Spodoptera frugiperda*]	1E-114	Partial
	c26837	606		6E-69	
	joint2_rep_c4921	492		3E-69	
	rep_c24787	1061		1E-99	
	c41348	512		2E-67	
