# Supplementary figures and images for: Establishment and analysis of a reference transcriptome for Spodoptera frugiperda
Source: BMC Genomics. 2014 Aug 23;15(1):704. doi: 10.1186/1471-2164-15-704 (PMC4150953; doi:10.1186/1471-2164-15-704)

## Number of reads per Illumina library

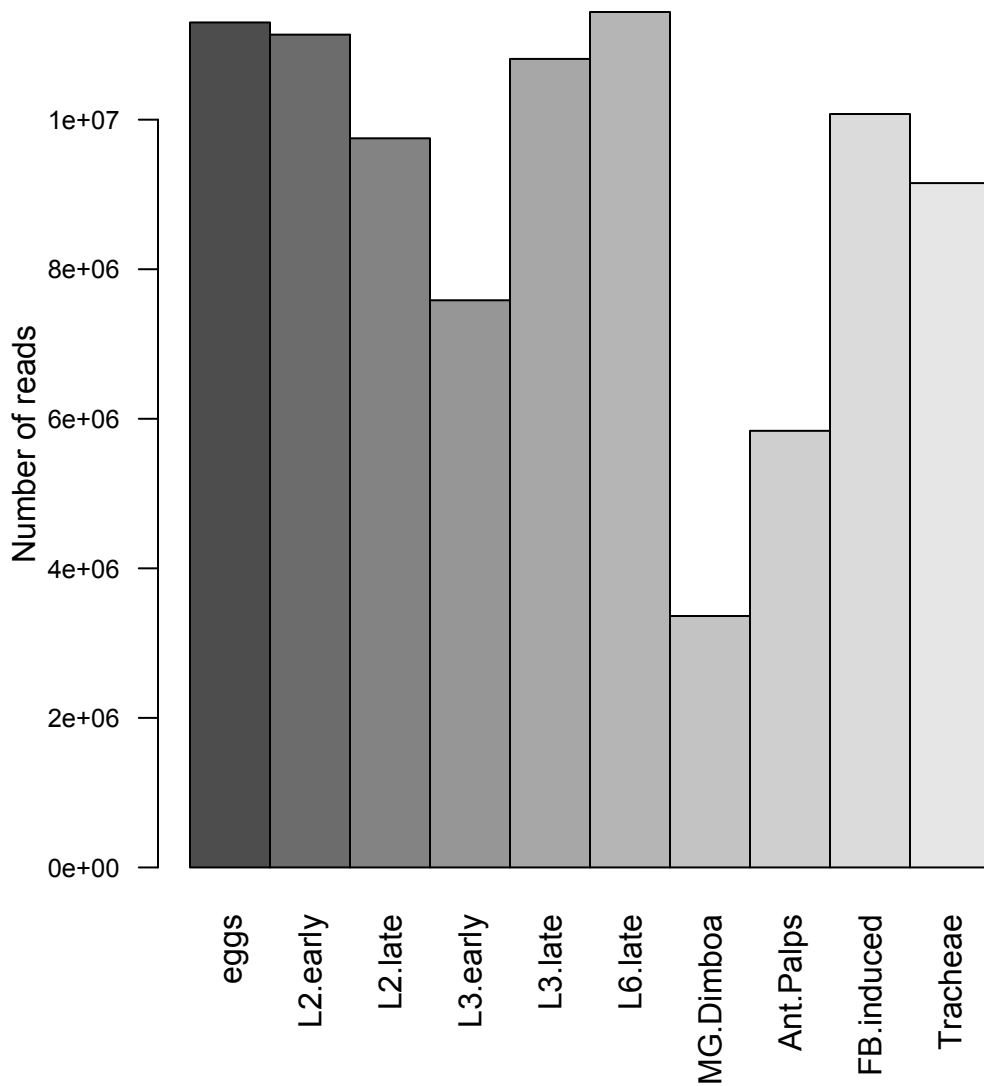

Supplement: Supplementary file 6 — Additional file 6: Table S5: Genes of Immunity. Innate immunity-related actors identified during S. frugiperda transcriptome wild analysis were classified in six groups. The first one contains transcripts encoding proteins involved in pathogen recognition as well as extracellular molecules associated to signal transduction. The second group contains proteins belonging to the Toll pathway which control among others the antifungal response and that also play a key role in developmental processes. The third group gathers proteins belonging to the Imd cascade that is at the center of the response against the Gram-negative bacteria. The fourth group lists the JAK/STAT pathway members; this pathway was originally identified through its role in embryonic segmentation, later, it was shown that this pathway is also involved in the innate immunity and stress response. The fifth group contains members of the JNK pathway. Finally, the sixth group made an inventory of all the effectors of the immune response. (PDF 138 KB) [file 12864_2014_6384_MOESM6_ESM.pdf]

A

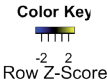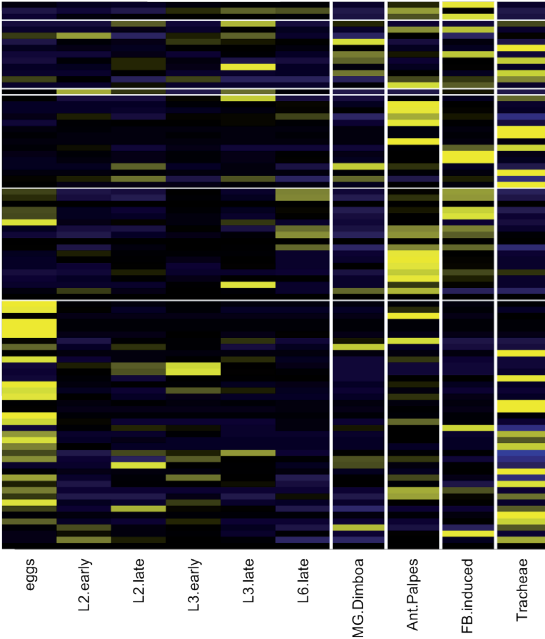

B

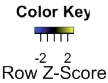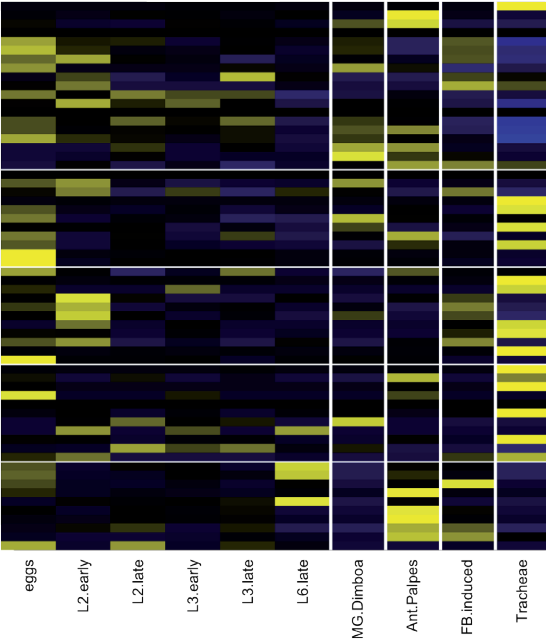

Supplement: Supplementary file 7 — Additional file 7: Figure S2: Expression time-course. Barplot showing the total number of reads for the 10 Illumina libraries from Table 1, column C. (PDF 774 KB) [file 12864_2014_6384_MOESM7_ESM.pdf]

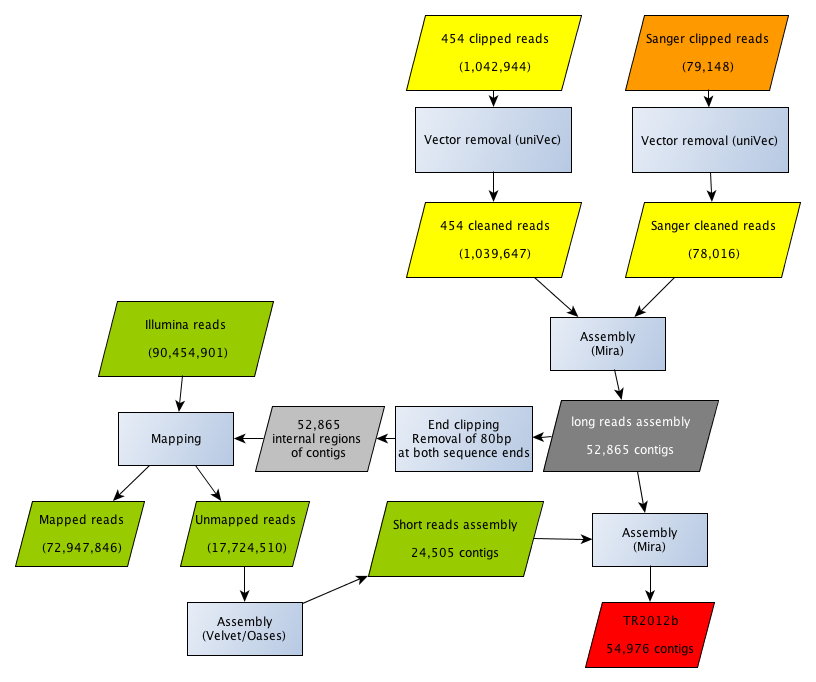

Supplement: Supplementary file 9 — Additional file 9: Figure S4: Expression of immunity genes. A - B. Heatmaps showing the expression as row scaled z-scores of S. frugiperda genes of immunity in the 10 Illumina RNAseq experiments from Table 1, column C. (PNG 60 KB) [file 12864_2014_6384_MOESM9_ESM.png]
