# Supplementary material for: Establishment and analysis of a reference transcriptome for Spodoptera frugiperda
Source: BMC Genomics. 2014 Aug 23;15(1):704. doi: 10.1186/1471-2164-15-704 (PMC4150953; doi:10.1186/1471-2164-15-704)
Supplement: Supplementary file 8 — Additional file 8: Figure S3: Candidate genes overexpressed in eggs. Heatmap showing the rpm normalized reads count of genes having more than 200 reads in eggs and less than 20 reads in L2e stage. (PDF 110 KB) [file 12864_2014_6384_MOESM8_ESM.pdf]

Number of contigs	54,976
Total size of contigs	36,925,829 nt
Longest contig	8,031 nt
Shortest contig	40 nt
Number of contigs > 500 nt	28,664 (52.1%)
Number of contigs > 1K nt	10,023 (18.2%)
Mean contig size	672 nt
Median contig size	520 nt
N50 contig length	876 nt
L50 contig count	12,672
contig %A	32.19
contig %C	17.83
contig %G	18.79
contig %T	30.78
contig %N	0.02
