# Supplementary material for: Establishment and analysis of a reference transcriptome for Spodoptera frugiperda
Source: BMC Genomics. 2014 Aug 23;15(1):704. doi: 10.1186/1471-2164-15-704 (PMC4150953; doi:10.1186/1471-2164-15-704)
Supplement: Supplementary file 10 — Additional file 10: Contains accession numbers for the BACs and qPCR primer sequences. (DOCX 71 KB) [file 12864_2014_6384_MOESM10_ESM.docx]

# Supplementary Information

## EBI BAC accession numbers

 FO681369        34G16_SfBAC_fin

 FO681370        74M02_SfBAC_fin

 FO681371        20M05_SfBAC_fin

 FO681372        52C06_SfBAC_fin

 FO681362        29O15_SfBAC_fin

 FO681373        72B06_SfBAC_fin

 FO681363        17L04_SfBAC_fin

 FO681364        15B23_SfBAC-fin

 FO681365        01O14_SfBAC_fin

 FO681366        20L07_SfBAC_fin

 FO681367        14A01_SfBAC_fin

 FO681368        75E05_SfBAC_fin

 FO681382        36K19_SfBAC_fin

 FO681383        74P15_SfBAC_fin

 FO681384        72H13_SfBAC_fin

 FO681385        28P14_SfBAC_fin

 FO681386        65A13_SfBAC_fin

 FO681374        96D18_SfBAC_fin

 FO681375        84K04_SfBAC_fin

 FO681376        83D20_SfBAC_fin

 FO681377        59C20_SfBAC_fin

 FO681378        66E15_SfBAC_fin

 FO681379        21I19_SfBAC_fin

 FO681380        51E05_SfBAC_fin

 FO681381        68E14_SfBAC_fin

## qPCR primers

| Sf_Cluster_GATC | Sf_TR2012b transcript | Forward primer | Reverse primer |
| --- | --- | --- | --- |
| lcl\|F67SK7T02F2ZPL_x1 | rep_c367 - eukaryotic translation initiation factor 3 subunit B | AGCAGCACACATTCTTGGTG | TGCATTCTGCCATACTTGGA |
| lcl\|F67SK7T02IJAZN_x2 | rep_c367 - eukaryotic translation initiation factor 3 subunit B | TCGAGAGACAGCCCTTCAAT | GAGTGAAGACACGCCAGTCA |
| lcl\|F67SK7T02HA500_x25 | rep_c559 - ribosomal protein L8 | GTGATTCGTGCTCAGCGTAA | ACCAGGGTCATGGATGATGT |
| lcl\|F67SK7T01DI0GR_x7 | joint2_rep_c6010 - nucleolar protein 58-like | GGGCTTCTTCAGTGGTGAAA | GCACTTCCTAGCTTGGCATC |
| lcl\|F67SK7T01BAWHH_x1 | joint2_c3760 - hypothetical protein (fend) | TTCAAGTGGAGCTTGTGTCG | CTGGTACACGTTCCCCTCTC |
| lcl\|F67SK7T02I3ND6_x12 | joint2_rep_c846 - CG17047 [D mel] | GCGGAGTCTTTGCTACTTGC | GGTTTCGAAGTCGATCCTCA |
| lcl\|F67SK7T01CW181_x3 | joint2_c3846 - homeobox protein Hox-D3-like - eve | CAGAGTGCCAAAGCTCTTCC | AGGTTGTGCCGGTATATTGC |
| lcl\|F67SK7T02ICK2H_x1 | joint2_rep_c1530 - uncharacterized protein | GTTCGTGAACAGGACCTTCC | TGGCTTTGATGATGTCGTGT |
| lcl\|F67SK7T01BMHI9_x2 | joint2_rep_c1530 - uncharacterized protein | CAGGCACGACATCATCAAAG | GCGCAAGAACCAGTTCATCT |
| lcl\|F67SK7T02JZX22_x1 | joint2_rep_c576 - cyclin B homolog | GGTGCTGGAGACATTCCACT | CCGACATCAGGAGCGTAGAT |
| lcl\|F67SK7T01BQ9X6_x3 | rep_c44869 - gametocyte specific factor 1-like | TTTTCCCATTTTCGTCTTCG | GGACAAGTCGTCTTGCCACT |
| lcl\|F67SK7T02JUOKN_x1 | No Hit - | TTCGGAAGAGAGTTCGGTTG | CATCTGCAGCAGCGAGAATA |
| lcl\|F67SK7T01BBAPI_x4 | joint2_rep_c1047 - gametocyte-specific factor 1-like | TTGGGTAGGGGATGGTGAT | GGCTCGAATAAACCAACCAA |
| lcl\|F67SK7T01DYRC3_x2 | joint2_c876 - histone deacetylase Rpd3 | TTGAATACTTCGGCCCTGAC | AGTGATGCGCTTGTCCTTTT |
| lcl\|F67SK7T02G8TQT_x1 | joint2_rep_c1747 - apoptosis inhibitor 5-like | AAGGTGCACTTACGGGAATG | GTTTCTTGCATTCAGCGACA |
| lcl\|F67SK7T02IMXZ5_x1 | c13387 - protein IMPACT | CGAAACGACAAGGTGGAACT | CCGTGGGAGACTTGGATTTA |
| lcl\|F67SK7T01A6Q7R_x1 | joint2_rep_c945 - uncharacterized protein | CTCCGACACCAGAAATCACA | TGCGTTCGAAGTCCTGAGTA |
| lcl\|F67SK7T01D0JEI_x1 | joint2_rep_c6159 - glycosyltransferase PglE | AGTGGAACGGTCGATACAGG | CAACAGACCAACGAGAATCG |
| lcl\|F67SK7T01A2221_x3 | joint2_rep_c7748 - No Hit | TACGGCGCTGTACTTCTCAA | CAGACACGTGACGGCTACAC |
| lcl\|F67SK7T02IVZNG_x38 | joint2_rep_c946 - cytochrome P450 333B11 | AAGCAAACAAGAGGCGAGAG | AACGGATTTGACGCCAATAA |
| lcl\|F67SK7T02I5XFT_x3 | joint2_rep_c846 - CG17047 (extracellular protein) | GATTGCCAAGTGAACCCAGT | TCTCGGTCACCAAGTTGTCA |
| lcl\|F67SK7T01A5JCN_x11 | joint2_rep_c1047 - gametocyte-specific factor 1-like | ACATGGGACTCGCATAAAAA | GGGCAGGGTTTTGTTTAAGA |
| lcl\|F67SK7T01A5JCN_x11 | joint2_rep_c1047 - gametocyte-specific factor 1-like | ACATGGGACTCGCATAAAAA | AGGGCAGGGTTTTGTTTAAGA |
| lcl\|F67SK7T02JEF8K_x6 | joint2_rep_c1647 - H2A histone family member V | TAAGGCTAAGGCCAAAGCAG | ACTCCAGAACCTCGGCTGTA |
| lcl\|F67SK7T02JDACH_x6 | rep_c15227 - imitation SWI | CTCGAGCGATACCACATCCT | GGCTTTCGGTTTAGGAGGAC |
| lcl\|F67SK7T02IMXZ5_x1 | c13387 - protein IMPACT | TGCCAAAGTGTTACCCTTCC | CCGTGGGAGACTTGGATTTA |
| lcl\|F67SK7T02IMXZ5_x1 | c13387 - protein IMPACT | CGAAACGACAAGGTGGAACT | CCGTGGGAGACTTGGATTTA |
| lcl\|F67SK7T01AZKRC_x1 | joint2_c2487 - CG42837 | TGTAAGGGCCACTGATACCC | AGACAGTGCCGGCGTTAG |
