# Supplementary material for: Establishment and analysis of a reference transcriptome for Spodoptera frugiperda
Source: BMC Genomics. 2014 Aug 23;15(1):704. doi: 10.1186/1471-2164-15-704 (PMC4150953; doi:10.1186/1471-2164-15-704)
Supplement: Supplementary file 1 — Additional file 1: Figure S1: Flowchart describing the Sf_TR2012b assembly process. (PDF 1 MB) [file 12864_2014_6384_MOESM1_ESM.pdf]

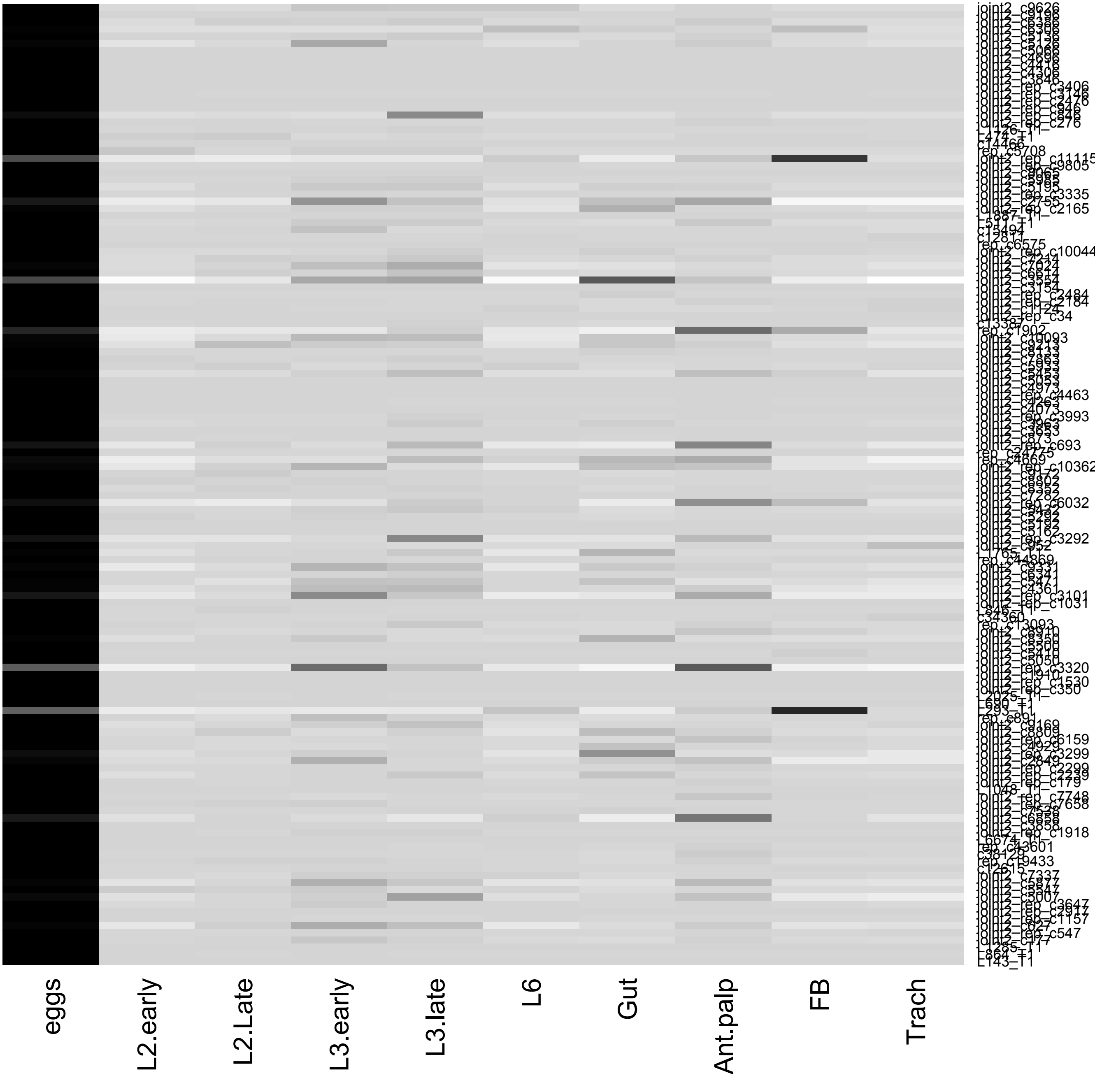

eggs
L2.early
L2.Late
L3.early
L3.late
L6
Gut
Ant.palp
FB
Trach
joint2_c9626
rep_c5708
joint2_rep_c3335
joint2_rep_c2165
joint2_rep_c34
joint2_rep_c4463
joint2_rep_c3993
joint2_rep_c693
joint2_rep_c3292
joint2_rep_c3101
joint2_rep_c3320
joint2_rep_c1530
joint2_rep_c350
joint2_rep_c6159
joint2_rep_c7748
joint2_rep_c7658
joint2_rep_c1918
rep_c19433
joint2_rep_c547
L143_T1
